# Optimizing Combination Therapies with Existing and Future CML Drugs

**DOI:** 10.1371/journal.pone.0012300

**Published:** 2010-08-23

**Authors:** Allen A. Katouli, Natalia L. Komarova

**Affiliations:** Department of Mathematics, University of California Irvine, Irvine, California, United States of America; Universidade de São Paulo, Brazil

## Abstract

Small-molecule inhibitors imatinib, dasatinib and nilotinib have been developed to treat Chromic Myeloid Leukemia (CML). The existence of a triple-cross-resistant mutation, T315I, has been a challenging problem, which can be overcome by finding new inhibitors. Many new compounds active against T315I mutants are now at different stages of development. In this paper we develop an algorithm which can weigh different combination treatment protocols according to their cross-resistance properties, and find the protocols with the highest probability of treatment success. This algorithm also takes into account drug toxicity by minimizing the number of drugs used, and their concentration. Although our methodology is based on a stochastic model of CML microevolution, the algorithm itself does not require measurements of any parameters (such as mutation rates, or division/death rates of cells), and can be used by medical professionals without a mathematical background. For illustration, we apply this algorithm to the mutation data obtained in [1], [2].

## Introduction

Chronic Myeloid Leukemia (CML) is a cancer of the white blood cells. It is characterized by the increased growth of predominantly myeloid cells in the bone marrow and the accumulation of these cells in the blood. The disease is associated with the Philadelphia chromosome, which arises by a reciprocal translocation between chromosomes 9 and 22 and harbors the BCR-ABL fusion oncogene [3]–[6]. The disease mostly affects adults, and its annual incidence is 1–2 per 100,000 people [7]; the only well-described risk factor for CML is exposure to ionizing radiation [8].

Small molecules that specifically target the BCR-ABL gene product provide a successful treatment approach which can lead to a reduction of BCR-ABL+ cells below detectable levels, at least during the early stages of the disease. The drug Imatinib has been mostly used in this respect [6]–[11]. It is the first member of a new class of agents that act by specifically inhibiting a certain enzyme that is characteristic of a particular cancer cell, rather than non-specifically inhibiting and killing all rapidly dividing cells. Imatinib has a number of side-effects, but in general is reasonably well-tolerated [9], compared to traditional chemotherapeutic agents, and it has not been found mutagenic [10].

As the disease advances, the chances of treatment failure rise due to the presence of drug resistant mutants that are generated mostly through point mutations [11]–[16]. Drug resistance can potentially be overcome by the combination of multiple drugs, as long as a mutation that confers resistance against one drug does not confer resistance against any of the other drugs in use. In addition to Imatinib, the drugs Dasatinib and Nilotinib are alternative inhibitors of the BCR-ABL gene product. Unfortunately, these three drugs exhibit a degree of cross-resistance because of one mutation (T315I) which confers resistance against all those drugs [1], [17]–[19]. In addition, there are more than 50 mutations that confer resistance against only one or two of the three drugs and not against the others [20].

Much research has recently been devoted to understanding the mechanisms of drug resistance in CML. Drugs in different combinations and different concentrations have been used in *in vitro* experiments to uncover the principles of resistance [21]–[26] and to suggest ways to avoid it. It has been suggested that using several drugs simultaneously, in a combination treatment, rather than sequentially, will improve the chance of treatment success by minimizing drug resistance [1], [27]. A promising goal is to come up with different inhibitors [28], and specifically, with agents that are effective against T315I mutants [2], [29]–[35].

In this paper we will formulate a mathematical model that allows for a systematic study of drug resistance in cancer and its effects on treatment. The model will utilize experimental data on the types of mutants that arise in the context of different treatments. The goal of this approach is to aid in optimal treatment strategy design. Our main result is a simple and intuitive algorithm of finding the optimal combination treatment which (1) minimizes the chances of treatment failure due to drug resistance, and (2) minimizes the number and concentration of the drugs used.

The basic mathematical model used here belongs to the tradition of stochastic modeling first created by [36]–[40] and continued by [41]–[43]. It is part of the larger effort to model anticancer therapies in general, and drug resistance in cancer specifically [44]–[58]. The approach developed in the present paper builds on our previous work, where we studied the stochastic dynamics of cell populations in the context of combination drug treatments [59], and created a framework to describe the phenomenon of cross-resistance [60]. Our goal is to make stochastic modeling of resistance in CML more relevant for practicing oncologists by helping them in making the best treatment protocol choices. To this end, we shift the emphasis from trying to calculate the probability of treatment success to a more practical issue of finding the combination of drugs that maximizes the chances of a successful treatment outcome. In this paper, we adapt the model to utilize experimental data by including information on different drug concentrations. Papers [1], [2] suggest that different concentrations of the three available drugs, imatinib, dasatinib, and nilotinib, can result in the outgrowth of different numbers of mutations. This means that resistance generation depends not only on the treatment composition, but also on the dosages of the various drugs. These data inspired us to revisit our modeling of combination treatments with a different approach.

We show that the probability of treatment success is (up to two significant digits) defined by the cross-resistant mutations. If the drugs in use possess a degree of triple cross-resistance (such as imatinib, dasatinib and nilotinib with the T315I mutation), then the presence of other mutations does not really alter the outcome. In general, the mutations which confer resistance to the largest number of drugs in the combination are the ones which define how likely it is that the protocol fails. Based on this concept, we developed a counting strategy which can weigh different treatment strategies according to their cross-resistance properties, and find the protocols with the highest probability of treatment success. This algorithm also takes into account drug toxicity by minimizing the number of drugs used, and their concentration. One useful feature of this algorithm is that it does not require measurements of any parameters (such as mutation rates, or division/death rates of cells), but relies entirely on the knowledge of the number and resistance types of mutants associated with each of the drugs in use.

The rest of this paper is organized as follows. First, we summarize and analyze the biological data which we use in our scheme. We then describe our analysis of the data, and calculate the number of mutations resistant to all possible combination treatments according to the number of drugs, their types and concentrations. We then present two algorithms to identify the best possible combination treatments. Finally, we apply both algorithms to the drugs studied in [1], [2] to find the best treatment strategies.

## Materials and Methods

In *in vitro* experiments described in papers [1], [2], CML cancer cells, Ba/F3 p210^bcr-able^ were exposed to a minimally cytotoxic agent, *N*-ethyl-*N*-nitrosourea (ENU), a potent inducer of point mutations. The cells were then cultured in 96-well plates supplemented with graded concentrations of inhibitors. After some time (about 28 days), wells with positive outgrows were expanded and then sequenced for mutations.

In [1], three different inhibitors, imatinib, dasatinib, and nilotinib, were used, in different combinations and solo. Inhibitor concentrations used for the three inhibitors are listed in Table 1. The noted concentrations were motivated by the fact that nilotinib is at least 20-fold and dasatinib at least 300-fold more potent than imatinib [1]. After analysis of the total of 768 wells, there were 726 mutations. Out of the 30 specific point-mutations that had been identified in imatinib resistant patients, 25 were recovered in this experiment. In total, 26 point-mutations were identified.

**Table 1:** Categorization of the doses of each inhibitor.

	Low Dose (*nM*)	Medium Dose(*nM*)	High Dose(*nM*)
**Imatinib**	2000	4000–8000	16000
**Dasatinib**	5	10–25	100–500
**Nilotinib**	50–250	500–1000	2000–5000
**SGX70393**	120–240	480–960	1920

The drug concentrations that were used in [1], [2] are all included in this table. We define our dose or concentration for each inhibitor (rows) through the doses used (columns).

In [2], an inhibitor of the T315I mutant SGX70393 was used both solo and in combination with the three inhibitors, imatinib, dasatinib and nilotinib. 27 different point-mutations were identified, 17 of which were novel in comparison to the ones recovered in [1].

### Stochastic modeling

*In vitro* experiments suggest that different concentrations of a drug give rise to different numbers and types of resistant mutants in treating CML. We will model this phenomenon by using an extension of the stochastic model for combination treatments with cross-resistance, first introduced in [59], [60]. The details of the model are presented in Text S1, Section 1, and here we only give a conceptual description.

**Figure 1:**
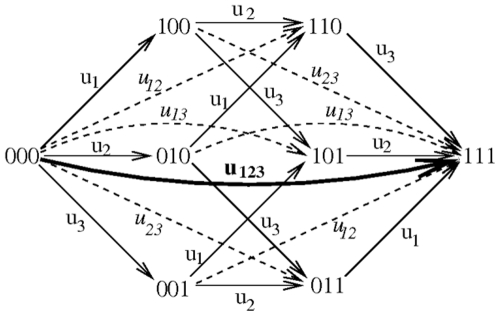
A mutation network for a three-drug treatment. The nodes correspond to different resistance phenotypes, and the arrows to mutation processes; mutation rates are marked next to the arrows. Singly-resistant mutations are denoted my solid lines, doubly-resistant mutations – by dashed lines and italic font, and the triply-resistant mutation by a thick line and bold font.

Stochastic dynamics occurs on a mutation diagram which specifies the mutation processes that create phenotypes resistant to various drugs, see figure 1. This network's nodes denote cancer cell phenotypes which have different characteristics with respect to their drug susceptibility. For example, if two drugs are used to treat the tumor, then potentially there could be at least four different cell types: those that are fully susceptible; we characterize those by the binary index *s = 00*; those resistant to drug 1 and susceptible to drug 2 (*s = 10*); those resistant to drug 2 and susceptible to drug 1 (*s = 01*), and those resistant to both drugs (or, fully-resistant), with *s = 11*. In general, if *m* drugs are applied in the course of the therapy, we have *2^m^* combinatorial resistance types. The binary index *s* has *m* positions corresponding to the *m* drugs; “1” in a given position denotes resistance to the corresponding drug, while “0” means susceptibility.

The nodes of the network are connected with arrows corresponding to mutation processes which transform one cell type into another. The mutation rates are marked by the arrows and denote the probability to produce one daughter cell of the transformed type upon a division of the cell of the given type. We neglect the back-mutations because they only provide a small correction to the processes governed by the forward mutations, see [61] and Section 2 in Text S1.

The dynamics of cells include the following events: a faithful cell division (such that both daughter cells are of the original type), a division with a mutation, and cell death (other events such as cellular quiescence and awakening from the state of quiescence could also be included, see [62], [63]). A division with a mutation implies that one of the daughter cells acquires a different phenotype, in agreement with the mutation network, while the other daughter cell does not carry the mutation. A simultaneous generation of two mutant cells (with mutations of a type relevant to the processes of interest) is possible, but it is a rare event compared to the production of only one mutant daughter cell, and will be neglected here.

The death rate of various resistance types consists of the natural death rate of cells (that is, their death rate in the absence of treatment), and the drug-induced death rate. To model the latter quantity, we ask: how do individual drug-induced death rates of several drugs interact under combination treatment conditions? In particular, what is the combined drug-induced death rate of two drugs applied in combination? On one extreme, it could be the same as the killing rate of the stronger of the drugs, which would mean that adding a weaker drug does not change the rate at which susceptible cells are killed. On the other extreme we have a sum of the two killing rates, which means that all drugs contribute proportionally to the killing rate. In the general case of *m* drugs, we assume that the effect of the drug combination on cell types susceptible to all the drugs is somewhere between the maximum (individual) killing rate and the sum of all the killing rates (see text S1 for the exact formulation).

At time *t = 0*, we assume that a cancerous colony starts growing stochastically from *M_0_* susceptible cells; at the time when treatment begins, there are *N* cells in the colony; this includes both susceptible cells and cells of other types generated before treatment starts. A stochastic model based on the processes described above has been formulated and analyzed in [59], [64]. In this paper we will move a step forward in terms of biological realism and design a way to incorporate the existing experimental data on BCR-ABL mutations into the model. One approach would be to list all the different molecular types (that is, take account of all the genotypes that appear in the experimental data) and keep track of whether they are resistant or susceptible to each of the drugs at each concentration. Consequently, there would be as many nodes in the network as there are different mutants. Furthermore, for each drug combination/concentration, different nodes would be subject to different drug-induced death rates. In particular, a given node can be susceptible for some drug combinations, and resistant for others.

However, here we adopt a different approach. We fix a simple combinatorial mutation network whose nodes have binary indices, as described above. These nodes correspond to different resistance phenotypes rather than genotypes. Depending on the drug combinations/concentrations that are used, the molecular types that comprise each phenotype will change. In order to capture the effect of drug concentration we note that as a result of an increase in a drug concentration, the relevant resistance classes will contain fewer mutants. In this model we assume that the total mutation rate between classes *i* and *j* is proportional to the number of different point mutations that transform a cell from class *i* to class *j*. Therefore, a decrease in a number of types comprising a resistance class will result in a decrease in the mutation rates generating this class. For example, an increase in the concentration of drug 1 (see figure 1) will reduce the resistance classes *100*, *101*, *110* and *111*, and therefore the rates *u_1_*, *u_12_*, *u_13_* and *u_123_* will be reduced.

### Classification of mutations

We will use the following convention for mutation rates:
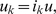
where 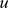 is the rate of point mutations per cell division per base-pair, and 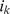 is the number of point mutations conferring resistance to the *k* ^th^ drug. For cross-resistance we use the same notation, using subscripts to indicate the particular drug numbers that the mutant is resistant to; for example, the number of mutations that confer resistance to drugs 1 and 3 is denoted by 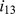. We will utilize the experimental data from papers [1], [2] in order to calculate the quantities *i_s_*, *i_sk_* and *i_skm_*. To this end, we develop some basic rules for data analysis.

We divide the concentrations of each drug into three categories: low dose, medium dose, and high dose; Table 1 describes these categories for each drug. From this convention, we can use the data to extract the types of point-mutations resistant to each drug, according to their category of concentrations. Table 2 lists all the mutation types found in the experiments and specifies if they are resistant to different drugs. This table indicates if a mutant is resistant to each concentration of each drug; this is marked by a “+”. If the mutant is susceptible to the concentration of a drug, then there are no markings in the table. In constructing table 2, we took the convention that if a mutation was found at a certain category, say medium dose, then we add this mutant to all lower categories, even if this was not found in the data (due to certain random fluctuations involved in any experimental procedures). The rationale behind this is as follows: if there was outgrowth of a particular mutant in presence of a drug with some concentration, then it is likely that this mutant can grow in any lower concentration of that drug.

**Table 2:** Possible mutations that may arise in the presence of inhibitors at different concentrations.

	I/L	I/M	I/H	N/L	N/M	N/H	D/L	D/M	D/H	S/L*	S/M*	S/H
M244V	+											
L248R	+	+		+	+		+			+	+	+
L248V	+			+			+			+		
G250E	+	+		+			+			+	+	+
Q252H	+	+	+				+	+		+	+	
Y253H	+	+	+	+	+					+	+	
E255K	+	+	+	+			+			+	+	
E255V	+	+		+	+		+			+	+	+
D276G	+											
E292V				+								
V299L							+	+				
F311I	+									+		
F311V	+											
T315I	+	+	+	+	+	+	+	+	+			
F317C							+	+		+	+	
F317I	+	+					+	+		+	+	
F317L	+						+	+		+	+	
F317V							+	+		+	+	+
M351T	+											
E355G	+											
F359C	+			+								
F359V	+											
V379I	+											
L384M	+			+								
L387F				+								
H396R	+	+										
G250W										+		
Y253F	+	+								+	+	+
Y253N	+									+	+	
G249D										+	+	
Q252E										+		
Q252H	+	+								+	+	
Y253C	+									+		
L248M										+	+	
L248Q	+	+								+	+	
F317S										+	+	
E258K										+		
G250V										+	+	
N322K										+		
E355G										+		
S417Y										+		
L248K	+	+								+	+	+
G250A	+									+	+	

*This indicates that this drug at this concentration cannot kill the un-mutated Native (or wild-type) cell.

The rows define the particular point-mutations and the columns define the inhibitors imatinib (I), nilotinib (N), dasatinib (D), and SGX70393 (S), with the indicated concentration (L, M, and H for Low, Medium and High, respectively). A “+” indicates that the mutant is resistant.

We use the data for combinations of inhibitors from both papers to identify mutants that were present in the different concentrations of the drugs in treatment. If a mutation is present in a combination of two drugs, then that mutant is resistant to each drug. For example, the mutant L248K was not recovered for solo treatments for imatinib or SGX70393. However, in combination this mutant did arise. Thus, we assume that this mutant confers resistance to both imatinib and SGX70393 individually according to their concentrations.

The data in Table 2 allows us to determine the number of resistant and cross-resistant mutants in the context of combining drugs at different concentrations. Among all the relevant mutations (that is, all the mutations that give rise to resistant phenotypes) in the context of combination treatments, we will distinguish three types:

1. Singly-resistant mutations, that is, mutations that confer resistance to only one drug (the number of mutations giving rise to resistance to drug *s* is denoted by the *i_s_* count).
2. Doubly-resistant mutations, that is, mutations that confer resistance to any two drugs (the *i_sk_* count).
3. Triply-resistant mutations, that is, mutations that confer resistance to three drugs simultaneously (the *i_skm_* count).

The values *i_s_*, *i_sk_*, and *i_skm_* are calculated from the experimental data described above, and are presented in Text S1, Section 2 and Tables 1, 2.1–2.4 and 3.1–3.8.

The stochastic model implemented here will be used for validating the counting algorithms designed in the next sections. All the parameters and their definitions are summarized in a table in Section 1 of Text S1. The parameter value ranges used in the simulations are as follows: the point mutation rate, *u*, is 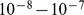 per cell division per base-pair, the cancerous population size at the beginning of treatment, *N*, is up to 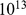 [65], the initial colony size 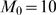, the death rate to division rate ratio is between *0* and *0.9*, and the drug-induced death rate to division rate ratio is between 1 and 10.

## Results

The stochastic model described here allows one to calculate the probability of treatment success, given the values of relevant parameters (such as the division and death of cells, mutation rates, etc). The problem is that at this moment we do not have reliable measurements of all the parameters available. Therefore, instead of attempting to attach a numerical value to the probability of treatment success, we will design an algorithm which allows us to select the best drug combination which maximizes the chances of successful treatment, while keeping the number and concentration of drugs as low as possible. It turns out that this is possible to accomplish without the knowledge of the parameters, but only based on the mutation information on various drugs at different concentrations. The algorithm is based on some fundamental properties of mutations which are described next.

### Mutation types and their influence on treatment success

In a three-drug treatment, there are three types of mutations (figure 1): singly- resistant mutations (the 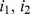, and 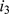 counts), doubly- resistant mutations (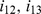, and 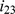 counts), and triply-resistant mutations (the 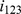 count). In order to see how much each type of mutations affects the probability of treatment success, we will turn “on” some of these mutations, while leaving the rest of them “off”. Numerical simulations (see the stochastic model of Text S1) show that triply-resistant mutations have a large influence on the probability of treatment success, whereas doubly- and singly-resistant mutations only give corrections to that probability of the order of 0.1% or less. In table 3, we show an example of this behavior by comparing the probability of treatment success in the presence and in the absence of singly- and doubly-resistant mutations. For singly-resistant mutations, we use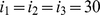, which is the maximum count that appears in Text S1, table 1. Similarly, for doubly-resistant mutants, we take 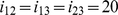 (compare to the values in tables 2.1–2.4 of Text S1). Finally, we let 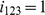, and compute the probabilities of treatment success for different tumor sizes with different combinations of mutations. In the body of the table, we present two probabilities corresponding to the two extreme values of the drug-induced killing rate, see inequality (7) of Text S1: the 1^st^ value in each cell corresponds to taking the maximum of the killing rates and the 2^nd^ one corresponds to taking the sum of the killing rates. We can see that switching partially-resistant mutations on and off only changes the probabilities by less than 0.1%.

**Table 3:** Probability of treatment success for a three drug combination treatment with different mutations “on” and “off”.

Log_10_N	Triply resistant mutations only	Doubly- and triply-resistant mutations	Singly- and triply-resistant mutations	All mutations
4.8	0.99930112 0.99934929	0.999300950.99934912	0.999301120.99934929	0.999300700.99934887
5.9	0.991122530.99172971	0.991119910.99172708	0.991122530.99172970	0.991116030.99172331
7.0	0.897091720.90350403	0.897061780.90347364	0.897091720.90350401	0.897017830.90343032
8.1	0.404998630.42232880	0.404919460.42224265	0.404998620.42232872	0.404803370.42211996
9.2	0.050465590.05400193	0.050449830.05398387	0.050465590.05400191	0.050426730.05395817
10.3	0.004132710.00443748	0.004131360.00443592	0.004132710.00443748	0.004129370.00443370
11.4	0.000323920.00034791	0.000323820.00034779	0.000323920.00034791	0.000323660.00034761

We take as parameters 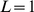, 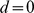, 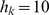, 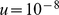, 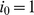, 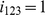, 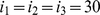, 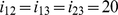 (for detailed definitions of the parameters see Text S1). For each tumor size and specific mutation gates we have two probabilities: the 1^st^ one corresponds to 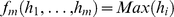, and the second one to 
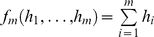
.

Using the basic model with all types of mutations on, and varying the number of triply-resistant mutations, 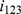, we calculated the probability of treatment success for different tumor loads, see table 4. Increasing the number of triply-resistant mutants, 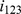, causes a significant decrease in the probability of treatment success. We conclude that the number of fully cross-resistant mutants dramatically affects the probability of treatment success. This implies that as long as there is at least one fully cross-resistant mutant, the success rate of a treatment solely depends on the number of these mutants, regardless of how many drugs are involved.

**Table 4:** Probability of treatment success for a three drug combination treatment with different number of triply-resistant mutations.

Log_10_N->	4.8	5.9	7.0	8.1	9.2	10.3	11.4
i_123_ = 0	1.000	1.000	0.9999	0.9988	0.9851	0.8376	0.2871
i_123_ = 1	0.9993	0.9911	0.8970	0.4048	0.0504	0.0041	0.0003
i_123_ = 2	0.9986	0.9824	0.8133	0.2538	0.0259	0.0021	0.0002
i_123_ = 3	0.9979	0.9738	0.7439	0.1849	0.0174	0.0014	0.0001
i_123_ = 4	0.9972	0.9654	0.6854	0.1454	0.0131	0.0010	0.0001

We take as parameters 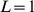, 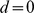, 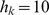, 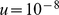, 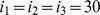, 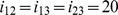, and 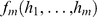 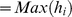 (for detailed definitions of the parameters see Text S1).

An analytical justification of these findings comes from an expansion, in terms of the small mutation rate, *u*, of the probability of treatment failure. In long-term drug combination treatments, the reason for treatment failure is assumed to be the creation of fully resistant mutants. The expected number of such mutants at the start of treatment (which is a deterministic quantity) correlates with the probability of treatment failure. Let us write down the system of deterministic equations governing the dynamics of all resistance classes; for illustration purposes we do this for the case of two drugs, and later generalize to three drugs:
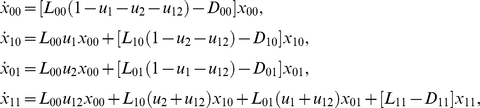
(2)where variables 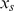 indicate the average numbers of mutants of resistance class *s*, *L_s_* and *D_s_* are the corresponding division and death rates, and the initial condition isw
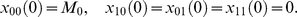
This system was derived by using standard methods from the stochastic master equation, see text S1. We are interested in the solution in the lowest order in *u*, therefore in the parentheses the mutation rates can be neglected compared to 1:
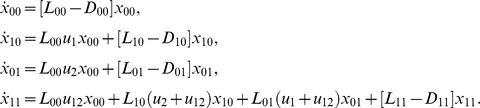
We can see that the quantity 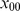 (fully-susceptible cells) is independent of the mutation rate. Quantities 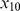 and 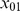 (one-hit mutants), in the leading order, are proportional to the first power on *u*. Finally, the quantity 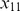 (fully-resistant mutants) in the leading order is proportional to the quantity 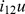, the rate of creation of doubly-resistant mutants directly from fully-susceptible mutants. In the absence of cross-resistance (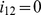), the expected number of fully-resistant mutants is proportional to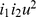.

Similarly, for three-drug treatments, the leading term in the expansion for the number of triply-resistant cells, 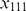, is proportional to 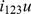. In the absence of triply-resistant mutants (that is, if 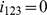), this quantity's largest contribution is quadratic in *u* and proportional to
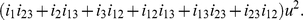
(3)Clearly, fully cross-resistant mutations comprise the dominant influence on the expressions for treatment failure (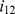 for 2-drug treatments and 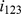 for 3-drug treatments); notice that the leading term in the expansion of the probability of treatment failure only has these mutations. Therefore, we can conclude that only these highest fully-cross-resistant mutations should be taken into account when evaluating the chances of treatment success for different drug combinations. This gives rise to some fairly straightforward algorithms which allow us to single out the most efficient treatment protocols. They are described in the next sections.

### Algorithm A1 for finding the best treatments in the case where there is at least one fully cross-resistant mutant

We will now develop an algorithm which allows us to identify the best possible treatments without the use of stochastic calculations. Our goal is to maximize the probability of treatment success, while minimizing the number and concentration of drugs.

In the case where there are triply-resistant mutations, the number of mutations that confer resistance to all the drugs in the treatment is of most importance in determining the best treatment strategy. Therefore, we only need to inspect tables 1, 2.1–2.4 and 3.1–3.8 of Text S1 for the best treatment strategies. The main idea is as follows. From all possible treatments, we need to identify the ones with the smallest number of fully cross-resistant mutants. Among these treatments, choose the ones that contain the smallest number of drugs at the lowest concentrations. More precisely, among treatments containing the same sets of drugs, we pick only the ones with the lowest concentrations, and if a particular treatment uses a subset of the drugs (at the same concentrations) of another treatment, we only include the treatment with the smaller number of drugs. The following algorithm (which we call Algorithm A1) executes this program and produces a set of the best possible treatments:

1. Identify all treatments that have the least number of fully cross-resistant mutants and list them, 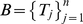. That is, all treatments in *B* will have the same number of fully cross-resistant mutants.
2. Divide *B* into three disjoint subsets: 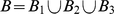, where 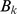 consists of treatments with *k* drugs. If the number of fully cross-resistant mutations is zero, stop and continue with Algorithm A2 in the next section. Otherwise, continue to step 3.
3. Note that 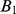 consists of one-drug treatments, each with a specific concentration. If a particular drug appears more than once in 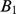 with different concentrations, then only keep the one with the lowest concentration, so that we have a refined set 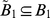.
4. If a drug with its particular concentration that is in the set 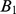 appears in 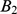 or 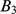, then do not consider those treatments. This will produce the first refinement of the sets 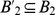 and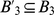.
5. If a pair of treatments in 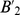 has the same two drugs and one of the drugs has the same concentration in the pair, then get rid of the treatment whose other drug is of a higher concentration. This will fully refine the set 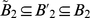.
6. If two of the drugs with specific concentrations in 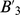 appear in 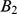, then get rid of it so to refine the set 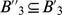.
7. Next, if a pair of treatments in 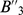 has the same three drugs, and the concentration of one or more drugs is higher in one treatment than in the other, then keep the treatment with the drug(s) of lower concentration. This will produce a fully refined set 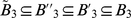.
8. The set 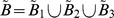 consists of the best treatment strategies.

For illustration, we will go through steps 1–8 of Algorithm A1 to identify the set of the best treatments possible with the three available inhibitors, imatinib, nilotinib, and dasatinib. Let us denote by
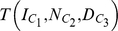
the treatment with imatinib *(I)*, nilotinib *(N)*, and dasatinib *(D)*. The subscripts, 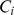, meaning concentration, will have four values: *0* for none, *L* for low, *M* for medium, and *H* for high. Thus, 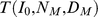, represents treating with twodrugs, nilotinib and dasatinib, both at medium concentrations.

We first note that any treatment with only one fully cross-resistant mutation is in the set *B*. We will turn our attention to 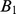.

This set consists of 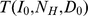 and 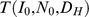, and is already fully refined, so that
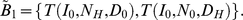
We next obtain the sets 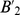 and 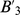 by getting rid of any two or three drug treatments that have either nilotinib or dasatinib at high concentrations:
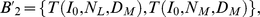


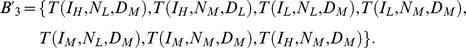
This completes steps 1–4. Now for step 5, we can refine 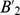 by noticing that both treatments have dasatinib at medium and nilotinib at low and medium. Thus, we have the fully refined set
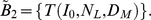
Next, we use 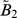 to refine 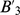:
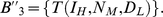
This set cannot be further refined and thus,
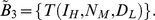
Thus, we have the following set of the best treatment strategies with imatinib, nilotinib, and dasatinib:
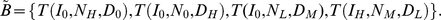
In words, the best treatments are as follows:

- 1 drug treatment with nilotinib at a high concentration.
- 1 drug treatment with dasatinib at a high concentration.
- 2 drug combination treatment with nilotinib at a low concentration and dasatinib at a medium concentration.
- 3 drug combination treatment with imatinib at a high concentration, nilotinib at a medium concentration, and dasatinib at a low concentration.

Since all these treatment protocols have a fully cross-resistant mutant, T315I, they all have similar probabilities. It will be the physician's decision influenced by the patient's needs that will determine exactly which treatment to use.

Next, we would like to consider incorporating the inhibitor SGX70393 in a combination treatment with at most three drugs.

### Algorithm A2 for finding the best treatments in the case where there are no fully cross-resistant mutants

If it is possible to use drugs which do not possess a possibility of triply-resistant mutations, this makes the probability of treatment success much higher. In this case, Algorithm A1 will not work, and treatment protocol optimization requires an alternative counting algorithm. This algorithm, which we call Algorithm A2, is developed in this section.

We first define two numbers as follows: for two drugs,
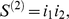
and for three drugs,
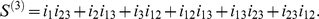
These choices are dictated by our theoretical considerations, see expression (3). It turns out that the quantities 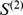 or 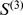 (for two- and three-drug treatments respectively) play a very important role in the ordering of various combination treatments. They indicate the main contribution to the probability of treatment failure (for two-drug and three-drug treatments respectively) due to resistant mutations. The smaller the 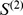 or 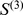 index, the larger is the probability of treatment success. In what follows we will show that the indices 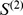 and 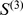 provide a convenient ordering of drug combinations equivalent to ordering in terms of their probability of treatment success.

**Figure 2:**
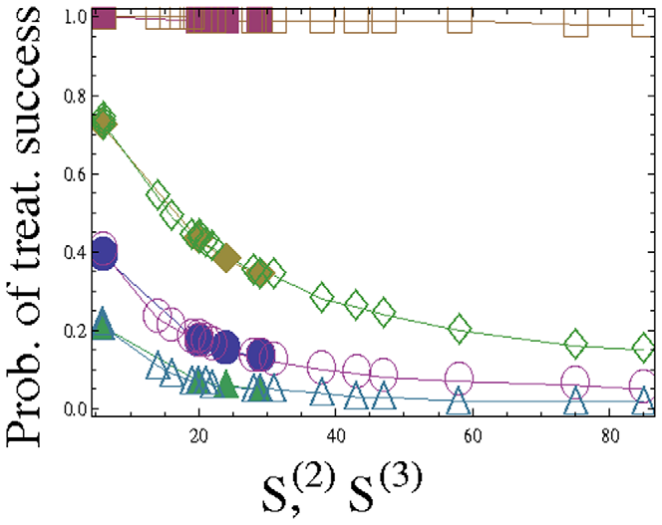
The probability of treatment success as a function of the numbers 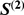 and 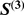. Different markers correspond to different treatment parameters: circles (division rate *L = 10*, death rate *d = 9*, drug-induced death rate *h_i_ = 10*, mutation rate *u = 10^−7^*, cancerous population size at the start of treatment *N = 10^10^*), squares (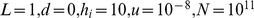), diamonds (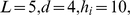 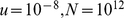), triangles (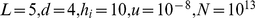). Empty markers denote three-drug treatments, and solid ones – two-drug treatments. The data are presented in tables 5 and 6.

To demonstrate this, we calculated the probabilities of treatment success using several different parameters, and we found that an increase in 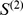 or 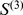 results in a decrease in the probability of treatment success, such that the numbers 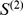 and 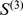 give an ordering of probabilities for any tumor load. In figure 2 we present the calculated probabilities of treatment success, for tumor load of size 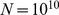, for different parameter values, for two-drug (solid markers) and three-drug (empty markers) treatments, as functions of the numbers 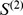 and 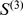 (see also tables 5 and 6).

**Table 5:** Set 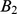 after step 3 of Algorithm A2.

	Concentrations of the drugs in the following order: Ima, Nilo, Dasa, SGX	S^(2)^	L = 10, d = 9, h_i_ = 10, u = 10^−7^, N = 10^10^	L = 1, d = 0, h_i_ = 10, u = 10^−8^, N = 10^11^	L = 5, d = 4, h_i_ = 10, u = 10^−8^, N = 10^12^	L = 5, d = 4, h_i_ = 10, u = 10^−8^, N = 10^13^
1	0, H, 0, H	6	0.3930	0.9985	0.7240	0.2075
2	0, 0, H, H	6	0.3930	0.9985	0.7240	0.2075
3	0, H, 0, M	20	0.1690	0.9947	0.4285	0.0700
4	0, 0, H, M	20	0.1690	0.9947	0.4285	0.0700
5	H, 0, 0, H	24	0.1460	0.9940	0.3830	0.0585
6	0, H, 0, L	29	0.1250	0.9922	0.3380	0.0485
7	0, 0, H, L	29	0.1250	0.9922	0.3380	0.0485

The second column shows the concentrations of each drug in order of imatinib (ima), nilotinib (nilo), dasatinib (dasa), and SGX70393 (SGX). The last 4 columns show the probability of treatment success given the parameters shown taking the sum of killing rates. For detailed definitions of the parameters see Text S1.

**Table 6:** Set 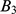 after Step 4 of Algorithm 2. Details are as in table 5.

	Concentrations of the drugs in the following order: Ima, Nilo, Dasa, SGX	S^(3)^	L = 10, d = 9, h_i_ = 10, u = 10^−7^, N = 10^10^	L = 1, d = 0, h_i_ = 10, u = 10^−8^, N = 10^11^	L = 5, d = 4, h_i_ = 10, u = 10^−8^, N = 10^12^	L = 5, d = 4, h_i_ = 10, u = 10^−8^, N = 10^13^
1	0, H, L, H	6	0.3980	0.9986	0.7400	0.2215
2	L, H, 0, H	6	0.4020	0.9985	0.7240	0.2205
3	L, 0, H, H	6	0.4020	0.9985	0.7240	0.2205
4	0, L, H, H	6	0.4030	0.9986	0.7410	0.2225
5	M, H, 0, H	6	0.4020	0.9986	0.7390	0.2205
6	M, 0, H, H	6	0.4020	0.9986	0.7390	0.2205
7	0, M, H, H	6	0.4030	0.9986	0.7275	0.2110
8	0, H, M, H	6	0.4030	0.9986	0.7285	0.2115
9	H, H, 0, H	6	0.4040	0.9986	0.7295	0.2125
10	H, 0, H, H	6	0.4040	0.9986	0.7295	0.2125
11	0, H, H, H	6	0.4040	0.9985	0.7295	0.2125
12	H, 0, M, H	14	0.2280	0.9966	0.5410	0.1055
13	H, M, 0, H	16	0.2050	0.9961	0.4890	0.0875
14	0, M, M, H	19	0.1800	0.9950	0.4440	0.0740
15	0, H, M, M	20	0.1740	0.9948	0.4340	0.0710
16	M, H, 0, M	20	0.1750	0.9947	0.4320	0.0705
17	M, 0, H, M	20	0.1750	0.9947	0.4320	0.0705
18	0, M, H, M	20	0.1760	0.9948	0.4350	0.0715
19	H, H, 0, M	20	0.1760	0.9948	0.4350	0.0715
20	H, 0, H, M	20	0.1760	0.9948	0.4350	0.0715
21	0, H, H, M	20	0.1760	0.9948	0.4360	0.0715
22	0, H, L, M	20	0.1730	0.9948	0.4325	0.0710
23	L, H, 0, M	20	0.1750	0.9947	0.4310	0.0705
24	L, 0, H, M	20	0.1750	0.9947	0.4310	0.0705
25	0, L, H, M	20	0.1760	0.9948	0.4340	0.0710
26	H, L, 0, H	21	0.1660	0.9945	0.4190	0.0675
27	H, 0, L, H	22	0.1590	0.9942	0.4070	0.0640
28	0, H, H, L	28	0.1340	0.9927	0.3530	0.0520
29	H, H, 0, L	29	0.1300	0.9924	0.3440	0.0500
30	H, 0, H, L	29	0.1300	0.9924	0.3440	0.0500
31	0, H, M, L	29	0.1290	0.9923	0.3435	0.0500
32	M, H, 0, L	29	0.1300	0.9923	0.3425	0.0495
33	M, 0, H, L	29	0.1300	0.9923	0.3425	0.0495
34	0, M, H, L	29	0.1300	0.9924	0.3440	0.0500
35	L, H, 0, L	29	0.1300	0.9923	0.3410	0.0490
36	L, 0, H, L	29	0.1300	0.9923	0.3410	0.0490
37	0, L, H, L	29	0.1300	0.9924	0.3435	0.0495
38	0, H, L, L	29	0.1290	0.9923	0.3425	0.0495
39	0, L, M, H	31	0.1200	0.9922	0.3380	0.0485
40	0, M, M, M	38	0.1010	0.9897	0.2795	0.0375
41	M, 0, M, H	43	0.0920	0.9891	0.2555	0.0330
42	0, M, M, L	47	0.0840	0.9872	0.2380	0.0305
43	L, 0, M, H	58	0.0700	0.9851	0.2010	0.0245
44	0, L, M, M	75	0.0550	0.9794	0.1620	0.0190
45	0, L, M, L	85	0.0490	0.9766	0.1455	0.0165

From this result, we construct an algorithm (Algorithm A2) for the case where there are no fully cross-resistant mutants. This algorithm will narrow down the sets 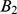 and 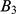 obtained from step 2 of Algorithm A1, to an ordered set, which starts with the treatment with the highest probability of success and lists the treatments in decreasing order; this set is also refined of treatments that have higher concentrations or more drugs involved than ones which produce the same probabilities. Here is the main idea of the algorithm. If there are treatments characterized by the absence of fully cross-resistant mutations, we arrange those treatments according to their indices 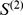 and 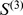. Within each subgroup (with a given index value), perform refinements identical to those of Algorithm A1. As a result, we obtain an ordered set of treatments which differ by their probability of treatment success. Below are the steps of Algorithm A2:

1. Suppose there are 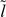 many treatments in set 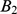 and the numbers 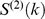 for the *k* ^th^ treatments in 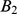 take the following distinct values: 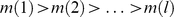, where 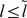. From this, we can partition the set 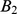 according to these numbers as follows: 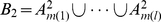.
2. Suppose there are 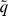 many treatments in set 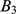 and the numbers 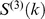 for the *k* ^th^ treatments in 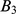 take exactly *q* distinct values, 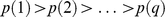, where 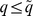. From this, we can partition the set 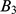 according to these numbers as follows: 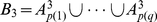.
3. We proceed to refine within each subset, 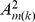 and 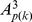. For the subset 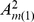 we identify all the sets 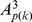, where 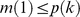, and perform steps 3–7 of Algorithm A1. Next for subset 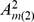 we identify all the sets 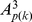, such that 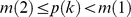, and perform steps 3–7 of Algorithm A1. Continue this process for all 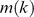, 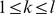. This will fully refine each subset of 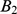 and 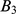; denote the new refined sets by
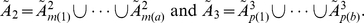

4. Suppose we order all possible numbers 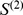 and 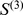 for the treatments in the sets 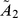 and 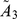, respectively, in increasing order: 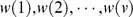. Then order all subsets 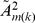 and 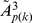 according to 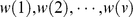, using the convention that if 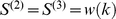, then we place 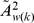 before 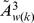. This will produce an ordered set, 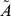, of the best treatments in sets 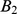 and 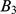, starting with the treatment with the highest probability of success.

The set 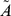 is an ordered set. A physician should consider the first treatment on the list; if the patient cannot tolerate that treatment, then the next treatment in the list should be considered, and so on. Note that this is different from the set 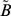, obtained from Algorithm A1. In set 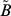, all treatments have the same success rate, give or take a percent. This is not true with the resulting set, 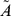, of Algorithm A2; there, the probabilities of treatment success can have a large range.

We will apply the new algorithm to obtain the best treatment strategies with the inclusion of the inhibitor SGX70393. Although the inhibitor SGX70393 is not available for use, it is a good example of a drug with no fully cross-resistant mutants if it is combined with any of the existing inhibitors.

We begin by identifying the sets 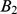 and 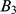 using step 1 of Algorithm A1. This produces 45 three-drug treatments and 7 two-drug treatments. After steps 3 and 4 of the algorithm we have tables 5 and 6 for the sets 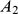 and 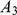, respectively. Notice that we also provide a probability of treatment success for several cases to show how well the numbers 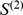 and 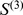 work in ordering the probabilities.

We proceed to step 5 by ordering the numbers 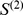 in decreasing order: 29, 24, 20, and 6 (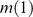, 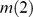, 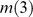, and 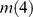).

For 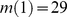, we perform steps 3–7 of Algorithm A1 on rows 6 and 7 of table 5 and rows 29–45 of table 6 which correspond to 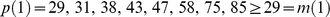. This step results in removing rows 29–38 of table 6, because they contain treatments which utilize the same drugs at the same concentrations as the treatments in rows 6 and 7 of table 5, together with additional drugs. This makes these treatments redundant.

For 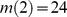, steps 3–7 of Algorithm A1 have to be performed on row 5 of table 5 and row 28 of table 6 which correspond to 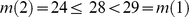. These steps do not lead to any further refinement.

For 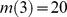, we perform steps 3–7 of Algorithm A1 on the rows 3 and 4 of table 5 and rows 15–27 of table 6 which correspond to 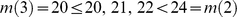. This step results in removing rows 15–25 of table 6.

Finally, for 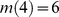, we perform steps 3–7 of Algorithm A1 on the rows 1 and 2 of table 5 and rows 1–14 of table 6 which correspond to 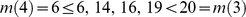. This step results in removing rows 1–12 of table 6.

We now order the remaining treatments according to step 7 and produce table 7.

**Table 7:** Set 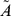 after all the steps of Algorithm A2. Details are as in table 5.

	Concentrations of the drugs in the following order: Ima, Nilo, dasa, SGX	S^(k)^	L = 10, d = 9, h_i_ = 10, u = 10^−7^, N = 10^10^	L = 1, d = 0, h_i_ = 10, u = 10^−8^, N = 10^11^	L = 5, d = 4, h_i_ = 10, u = 10^−8^, N = 10^12^	L = 5, d = 4, h_i_ = 10, u = 10^−8^, N = 10^13^
1	0, H, 0, H	6	0.3930	0.9985	0.7240	0.2075
2	0, 0, H, H	6	0.3930	0.9985	0.7240	0.2075
3	H, 0, M, H	14	0.2280	0.9966	0.5410	0.1055
4	H, M, 0, H	16	0.2050	0.9961	0.4890	0.0875
5	0, M, M, H	19	0.1800	0.9950	0.4440	0.0740
6	0, H, 0, M	20	0.1690	0.9947	0.4285	0.0700
7	0, 0, H, M	20	0.1690	0.9947	0.4285	0.0700
8	H, L, 0, H	21	0.1660	0.9945	0.4190	0.0675
9	H, 0, L, H	22	0.1590	0.9942	0.4070	0.0640
10	H, 0, 0, H	24	0.1460	0.9940	0.3830	0.0585
11	0, H, H, L	28	0.1340	0.9927	0.3530	0.0520
12	0, H, 0, L	29	0.1250	0.9922	0.3380	0.0485
13	0, 0, H, L	29	0.1250	0.9922	0.3380	0.0485
14	0, L, M, H	31	0.1200	0.9922	0.3380	0.0485
15	0, M, M, M	38	0.1010	0.9897	0.2795	0.0375
16	M, 0, M, H	43	0.0920	0.9891	0.2555	0.0330
17	0, M, M, L	47	0.0840	0.9872	0.2380	0.0305
18	L, 0, M, H	58	0.0700	0.9851	0.2010	0.0245
19	0, L, M, M	75	0.0550	0.9794	0.1620	0.0190
20	0, L, M, L	85	0.0490	0.9766	0.1455	0.0165

**Figure 3:**
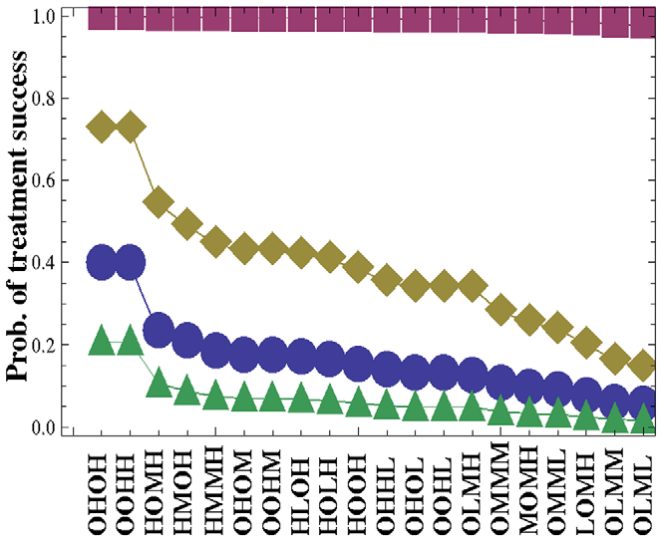
The ordered set of the best treatment protocols resulting from a application of Algorithm A2. The probability of treatment success is plotted as a function of treatment protocols, see also table 7. The parameters are as in figure 2.

Algorithm A2 allowed us to narrow down the 57 treatments of step 2 to just 20 treatments. These 20 treatments are in order of decreasing probability of treatment success. They are presented in figure 3, as a plot of the probability of treatment success as a function of different treatment protocols. As we can see, all the best treatment protocols rely on the usage of the T315I inhibitor. Furthermore, the treatments corresponding to the highest success probabilities are two-drug treatments where both drugs are used at the highest concentrations. These are followed by three-drug treatments with drugs used at lower concentrations.

## Discussion

We have developed a counting method to narrow down all possible treatments to the best ones. Although the development of the methodology relies on stochastic calculations, this counting method can be used by biologists and physicians, and does not require a strong mathematical background. To implement the method, one does not need to calculate the specific probabilities for each treatment, but simply follow the steps to select and order different protocols. Along with the counting scheme, which accounts for the hierarchy of probabilities of success, we weed out many treatments to minimize the number of drugs in combination and their respective concentrations.

To create this method we used the data from biological experiments to identify which types of point mutations can cause resistance to various drugs at different concentrations. In general, in the context of multi-drug treatments, we classify all possible mutations into three classes, singly-, doubly-, and triply-resistant mutations, depending on how many different drugs (out of the three drugs in the combination) they confer resistance to. From the experimental data, we count the numbers of mutations of each type, for each possible treatment. From this information, we provide two algorithms: one that deals with treatments that do not possess any triply-resistant mutants (Algorithm A2), and another one for treatments which include only drugs with a possibility of triply-resistant mutations (Algorithm A1).

The mathematics that we used to develop these methods included a stochastic model of resistance [59], [60] refined to allow for different drug concentrations. We used analysis on this model along with numerical results to support the proposed counting techniques. One important pattern that we found is that in the presence of a possibility of triply-resistant mutations, other types of mutations (such as doubly-and singly-resistant mutations) do not make a noticeable difference in the probability of treatment success. This result suggests that in choosing the optimal combination treatment, one should look for drugs with the smallest number of fully cross-resistant mutants. If for a three-drug therapy, a triply-resistant mutant exists (which is the case with imatinib, dasatinib and nilotinib), then the presence of other mutations (which may change depending on the dosage of the drugs) does not make a difference for the probability of the treatment success. Therefore, one should use the lowest possible doses (and the smallest number of drugs) as long as there is only one fully-cross-resistant mutant present (note that lowering the doses too much would lead to a possibility of more than one triply-resistant mutants). This result is not in contradiction with the recent work of [66], where it is suggested that once imatinib-based therapy failed, it is possible to find out what mutants caused resistance, and then choose the best second-line drug based on this. The knowledge of the mutations in an individual patient will of course help refine the treatment strategy. Our approach only gives a suggestion about the best plan of action before we know anything about the mutations in an individual patient.

The algorithms of treatment optimization developed here have the advantage that they do not require any information on the (usually unknown) parameters which are part of traditional stochastic modeling. We do not need to know the tumor size, the mutation rates, the growth/death/quiescence rates of cancer cells, or the killing rates of individual drugs or drugs in combination. The only information which is required to execute the algorithm is the activity spectra of the drugs in use. These are comprised of data on the numbers and resistance properties of mutants resistant to each of the drugs. We hope that this technique will aid physicians in the choice of the best possible combination therapies with current and future, undeveloped inhibitors, which maximize treatment success and minimize the harm that a patient may endure from side effects of such drugs.

In this study, we illustrated the usage of the algorithms with the data from [1], [2]. The method developed here is rather general and can be applied to other data sets. An example of a recently built data set which includes mutations in the context of imatinib, dasatinib, nilotinib and a newer drug bosutinib, can be found in [67]. A very promising new drug which shows activity against T315I-mutants is danusertib, whose properties are now being studied [31], [34], [35], [68]. Once more information is available on the activity spectrum of this drug, one will be able to use Algorithm A2 in treatment designs involving danusertib together with some of the older generation inhibitors. In this case, no triply-resistant mutants exist, and one can come up with a hierarchy of combination protocols based on the doubly- and singly-resistant mutations.

In the present study we concentrated on combination treatments. Although the common present clinical practice is to treat patients with one drug (usually imatinib) and if resistance arises, switch to a different drug, it has been suggested that a more efficient treatment strategy is to combine several drugs [1], [12], [27]. Combination protocols have the advantage of minimizing the chance of treatment failure due to drug resistance generation. It can be shown by means of mathematical modeling that cyclic therapies (which consist of cycles of single-drug applications) are not nearly as efficient as combination therapies at achieving the maximum treatment success. These considerations provided motivation for optimizing combination protocols on the basis of cross-resistance and drug concentration data. A similar analysis of cyclic therapies, and also of informed therapies where certain aspects of individual patient mutation spectrum are known, is a subject of current and future research.

Finally, a very desirable future extension of the present study would be to apply the algorithm to *in vivo* data when those become available. For that more clinical trials must be conducted with combination treatments consisting of imatinib, nilotinib, dasatinib and any other drugs that are developed, at different levels of concentration.

## Supporting Information

Text S1 (2.61 MB DOC)
