# Supplementary material for: Optimizing Combination Therapies with Existing and Future CML Drugs
Source: PLoS One. 2010 Aug 23;5(8):e12300. doi: 10.1371/journal.pone.0012300 (PMC2925944; doi:10.1371/journal.pone.0012300)
Supplement: Text S1 — (2.61 MB DOC) [file pone.0012300.s001.doc]

# Text S1

**1. The basic stochastic model**

To set up the stochastic description of cell dynamics, we denote by *j_s_* the number of cells of resistance class *s*. Let denote the probability that at time *t* there are *j_s_* cells of resistance class *s*, for all classes *s,* with $0\leq s\leq n=2^{m}-1$. Suppose that cancerous cells divide with rate *L_s_*, and die with rate *D_s_*. We assume an exponential time-distribution for various events, and set up the following Kolmogorov forward equation for a linear birth-death process: with

here, $u_{s\to k}$ denotes the mutation rates for all arrows originating at *s*, see the mutation network of the resistance types. This equation can be used to derive equations for the moments. For example, if we need to obtain an ODE for the time-evolution of the expected number of fully-susceptible cells, we multiply the Kolmogorov forward equation by *j_0_* and perform summation over all the indices. System (2) of the main text was obtained in this way in the case of m=2 drugs. There, the expected number of fully susceptible cells is given by , and similarly for the other average quantities.

The mutation diagram for the case of m=3 drugs is presented in figure 1 of the main text. Technically, not all the mutation rates are marked on that figure. For example, the arrow connecting type 100 and type 110 must have the total mutation rate *u_2_+u_12_*, because mutations conferring resistance to both drugs 1 and 2 will enter in the total rate of generating type 110 from type 100. However we chose to omit those additional rates in figure 1 because they would make the figure hard to read. We did include these rates in figure 1 of text S1 for *m=2* drugs.

*1.1 The probability generating function and the method of characteristics*

The stochastic model described above was analyzed by using the filtered Poisson approximation (also known as a doubly-stochastic process) by Goldie and Coldman (1983). Under some assumptions, this method allows one to obtain an analytical solution for quantities of interest. In this paper we do not aim to obtain an analytical solution, but rather to extract a simple counting scheme out of the full stochastic process. To do this, we use the following probability generating function: with $\vec{\xi}=(\xi_{0},\ldots,\xi_{n})$. This function satisfies a partial differential equation (obtained by standard methods from the Kolmogorov forward equation above):

$$\frac{\partial\Psi}{\partial t}=\sum_{s=0}^{n} \frac{\partial\Psi}{\partial\xi_{s}}\left(L_{s}(1-\sum_{k} u_{s\to k})\xi_{s}^{2}+\left[\sum_{k} L_{s}u_{s\to k}\xi_{k}-(L_{s}+D_{s})\right]\xi_{s}+D_{s}\right).$$

Following the standard technique for hyperbolic equations, we obtain a system of equations for characteristics:

where

$$F_{s}(\vec{\xi};t)=L_{s}(1-\sum_{k} u_{s\to k})\xi_{s}^{2}+\left[\sum_{k} L_{s}u_{s\to k}\xi_{k}-(L_{s}+D_{s})\right]\xi_{s}+D_{s}.$$

The dependence on time in the right hand side comes from the fact that the kinetic rates such as birth and death rate can in general be functions of time. In order to evaluate the function $\Psi(\vec{\xi}';t')$ at some point$\vec{\xi}'=(\xi'_{0},\ldots,\xi'_{n})$, we ``reverse the time” by the change of variables, $t\to t'-t,$ and solve the following system:

(1)

Then the desired function is given by

$\Psi(\vec{\xi}';t')=\prod_{s=0}^{n} [\xi_{s}(t')]^{M_{s}},$ (2)

where in the right hand side the functions $\xi_{s}(t)$ are solutions of the above system and the constants *M_s_* are the initial abundances of mutants of type *s*. We will assume for simplicity that initially, there are *M_0_* cells of type *s=00*, and zero cells of any other types.

*1.2 Treatment strategies, drug-induced death rates and symmetry assumptions*

In the particular case of *m=2* drugs and four mutant classes (see Figure 1), we have the following four characteristic equations:

(3)

(4)

(5)

(6)

where the time-dependence of the coefficients is implicit. A similar system can be written for a general *m*-drug treatment.

**
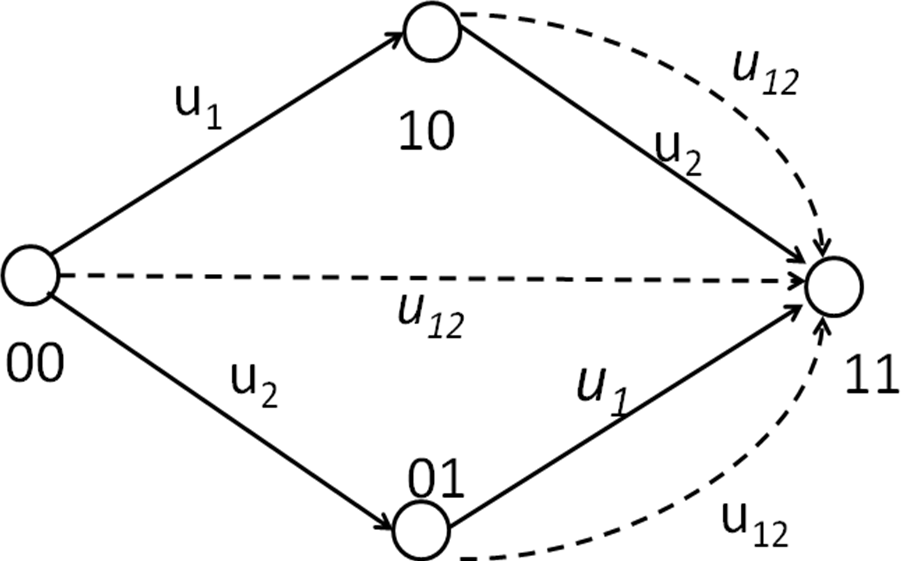
**

**Figure 1.** *A mutation diagram for the case m=2.* The mutation rates of the four types are indicated.

The death rates of various types consist of the natural death rate of cells, *d_s_*, (that is, their death rate in the absence of treatment), and the drug-induced death rate, *H_s_*:

$$D_{s}=d_{s}+H_{s}$$

To model the function *H_s_*, we will use the following considerations. Let us denote the *k*^th^ drug killing rate by $h_{k}.$ This quantity measures the drug-induced death rate of susceptible cell types when drug *k* is applied. The drug-induced death rates of various phenotypes will be a function of the quantities $h_{k}.$ For an *m*-drug combination treatment, let us denote by $f_{m}(h_{1},\ldots,h_{m}),$ the drug-induced death rate for a fully susceptible cell. This quantity satisfies the inequality

$Max(h_{i}:i=1, 2,\ldots,m)$ $\leq$ $f_{m}(h_{1},\ldots,h_{m})$ $\leq$ $\sum_{i=1}^{m} h_{i}$. *(7)*

In other words, we assume that the effect of the drug combination on cell types susceptible to all the drugs is somewhere between the maximum (individual) killing rate and the sum of all the killing rates. For the cells resistant to drug *k*, we have $h_{k}=0,$ so that

$$f_{m}(h_{1},\ldots,h_{k-1},h_{k},h_{k+1},\ldots,h_{m})=f_{m}(h_{1},\ldots,h_{k-1},h_{k+1},\ldots,h_{m}).$$

For example, for *m=2* we have:

$$\begin{matrix} H_{00}=f_{2}(h_{1},h_{2}),\begin{matrix} & \end{matrix}H_{10}=f_{2}(h_{2})=h_{2}, \\ H_{01}=f_{2}(h_{1})=h_{1},\begin{matrix} & \end{matrix}H_{11}=f_{2}(0)=0. \end{matrix}$$

We will consider both limiting values in inequality *(7)* and keep in mind that they provide the lower and the upper bound for $f_{m}$. A scenario that is not included here is a possible synergistic effect of the drugs, where the combined action of several drugs is stronger than the sum of individual actions. However, as we show below, the particular form of the function $f_{m}$ does not play a significant role, and therefore we conjecture that a presence of a degree of synergy will not change our results.

The functions *h_k_ (t)* defined here depend on the particular strategy used. As different drugs are applied, the ``strength'' of each drug, which depends on the concentration of the drug in the patient's blood, changes as some smooth function of time. In this paper we simplify this picture by assuming that the functions *h_s_ (t)* are piecewise constant: they are assumed to have a constant nontrivial value for all the susceptible classes as long as the patient is treated with a given drug, and they become zero after the drug is discontinued.

To summarize, we have the following convention for the cells’ death rates: $D_{s}=d_{s}+H_{s}=d_{s}+f_{m}(h_{1},\ldots,h_{m})$, where the argument of f contains only the killing rates of the drugs to which the type *s* is susceptible. A simplifying symmetry assumption that can be made in the absence of more information on the division and death rates of various types is that they are all equal to each other, that is, *L_s_=L* and *d_s_=d*.

*1.3 The probability of treatment success*

The stochastic model will be applied to a disease which starts at time *t=0* with *M_0_* fully susceptible cells, and proceeds until time $t_{*}$, at which point a combination treatment starts. In our studies, we will assume that the colony size at the start of treatment, *N*, and the time when treatment begins,$t_{*}$, are related by means of a deterministic equation for the average population size. In particular, if the growth and death rates of all the resistant types are the same in the absence of treatment, that is, if *L_s_=L* and *d_s_=d*, we have $t_{*}$*=*log*(N/M_0_)/(L-d).* In all the numerical simulations we will take $M_{0}=10$unless noted otherwise.

The main objective of the stochastic model is to calculate the probability of treatment success, which is the same as the probability of extinction of the colony. This quantity is given by

To evaluate this function, we will use general formula (2) with *M_s_=0* for all (partially) resistant types:

$$\Psi(\vec{0};t)=\xi_{00}^{M_{0}}(t),$$

where $\xi_{00}$*(t)* is the solution of system (3-6) with the initial conditions

$\xi_{00}=\xi_{01}=\xi_{10}=\xi_{11}=0$ (8)

To exclude the scenarios where the cancerous colony goes extinct spontaneously before treatment starts, we will be studying the following slightly modified quantity:

$P_{success}(t)=\frac{\xi_{00}^{M_{0}}(t)-(d/L)^{M_{0}}}{1-(d/L)^{M_{0}}},$ (9)

where we assumed that the division and death rates of all types are equal before treatment starts, *L_s_=L* and *d_s_=d*. To obtain solution $\xi_{00}$*(t)*, system (3-6) (or a similar system for more than two drugs) with piecewise-constant coefficients can be solved numerically. The time-dependence of the coefficients of this system is a consequence of having two separate stages in the microevolution of the colony: before the treatment starts, and during treatment. In the first, pre-treatment, phase, the death rates of all the types are simply given by their natural death rates, *d*. After the treatment starts, the death rates of all the types may be different, depending on their resistance properties. In practical terms, this means that we solve the system for characteristics (e.g. system (3-6) in case of two drugs) twice, with pre-treatment and during-treatment coefficients. For more details we refer to our earlier work, Komarova (2006).

The limiting value of the probability of treatment success,

$\lim_{t\to\infty} P_{success}(t)$, (10)

is of a particular interest. This approximation corresponds to long-term treatment strategies where the drugs are used long enough for all the susceptible types to be eliminated with a high probability. In general, long-term strategies are defined as treatments for a time *t* which is long enough to reduce the expected number of susceptible mutants to less than a unity. A characteristic time for such a process is given by log*N/(D_0_-L)* , where *D_0_* is the total death rate of fully-susceptible cells under the given treatment. When validating the counting algorithm, we used both formulas (10) and (9) with the assumption that the treatment time is long, in the sense described here.

The probability of treatment success, equations (9) and (10), depends on all the parameters of the system. The parameters are summarized in the following table:

| Notation | Definition | Notation | Definition |
| --- | --- | --- | --- |
| *L_s_ (L)* | Division rate of type *s* cells (of all cells if equal) | *u* | Point mutation rate (per cell division per base-pair) |
| *D_s_* | Total death rate of type *s* cells | *u_s_*, *u_sl_*, *u_slk_* | The mutation rates at which resistance to the given drugs is generated, per cell division |
| *d_s_ (d)* | Natural death rate of type *s* cells (of all cells if equal) | *i_s_*, *i_sl_*, *i_slk_* | The number of mutations conferring resistance to the given drugs |
| *H_s_* | Drug-induced death rate of type *s* cells | *N, t** | The number of cancerous cells at the moment when treatment starts (*t**). |
| *h_k_* | The killing rate of drug *k* applied solo | *M_0_* | Initial number of susceptible cells, as the colony starts growing |

While the knowledge of parameter values is necessary to obtain the numerical value for the probability of treatment success (as given by our stochastic model), the coefficient values are not required for the counting algorithm developed in this paper.

**2. The special model**

The necessity to create a special model comes from the following observation. From Table 2 of the main text we can see that the inhibitor SGX70393 at low or medium concentrations cannot kill the wild-type cells.

Recall that in the basic model for *m*- drug treatments, we have *2^m^* different resistant types, each characterized by their susceptibility to each drug; the types are arranged in a directed network with the first node (the one to which no arrow points) being the fully-susceptible type. The initial condition is the existence of $M_{0}$ native (or wild-type) cells, which are fully-susceptible to all the drugs. Since SGX70393 cannot kill wild-type cells, the native type is no longer susceptible to all the drugs, and therefore the basic model no longer applies. This is only the case for treatments with SGX70393 at low and medium concentrations. To describe these two scenarios, we build the following modified model.

Define $i_{0}$ to be the number of mutants that SGX70393 can kill (this number is different for low and medium concentrations of SGX70393, and also depends on the number of drugs used in combination with SGX70393, see Section 3 below). Define a fully susceptible cell type 0*, 00*, or 000* in a 1, 2 or 3 drug treatments with SGX70393, respectively (figure 2). In this special case, the values of drug-induced death rates for phenotypes 0, 00, or 000 are the same as the phenotypes 1, 01, or 001 (in the 2 or 3 drug treatments we take SGX70393 to be the last drug), however, the former are wild-types and the latter are mutated cell types. Figure 2 presents the diagrams of a 1 and 2 drug treatment of this special model. It shows all the relevant mutation rates, as well as the drug-induced death rates for all resistance types; those are denoted as *H_s_*, such that the total death rate of type *s* is given by $D_{s}=d_{s}+H_{s}$*.*


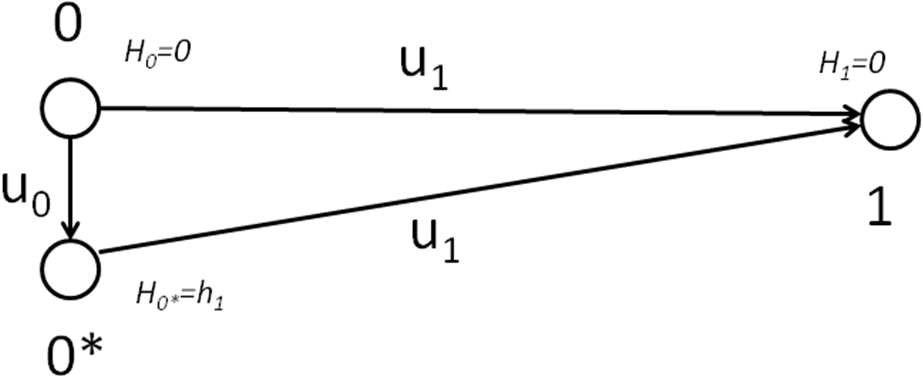


**One Drug**


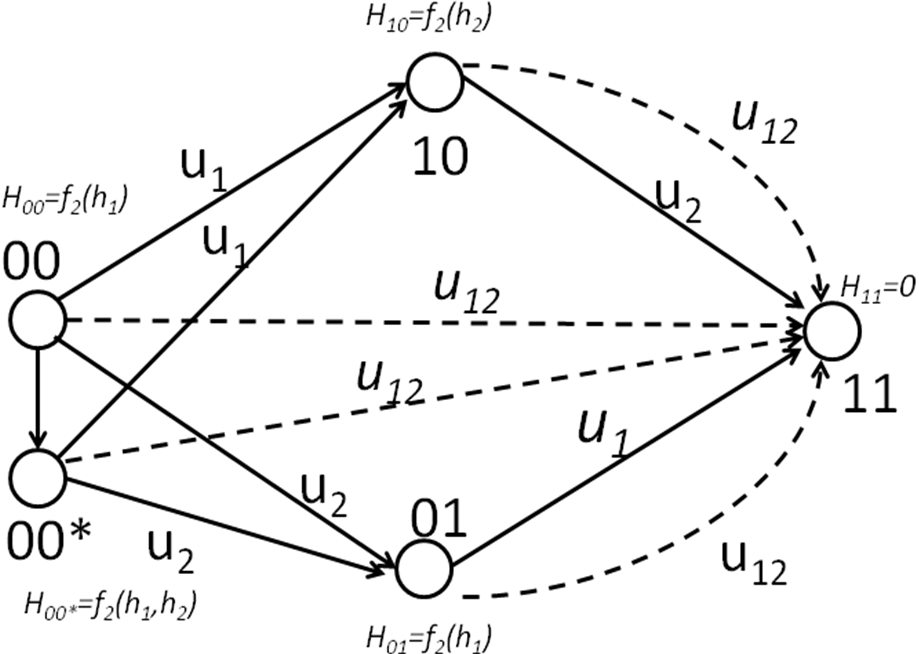


**Two Drugs**

**Figure 2.** *Diagrams for the* *special model for a one or two drug combination treatment with the inhibitor SGX70393.* In the two drug treatment, we take SGX70393 as the second drug. The drug-induced death rates for all the resistance types are shown, they are denoted by *H_s_*, such that the total death rate of type *s* is given by $D_{s}=d_{s}+H_{s}$*.*

Applying the method described in Section 1, we construct systems of characteristic equations in the case of one-, two- and three-drug treatments in the presence of inhibitor *SGX70393* at low or medium concentrations. For one-drug treatments we have:

For two-drug treatments we have:

Finally, for three-drug treatments we have (using the integer representation of the binary number):

We use the drug-induced death rate which appears in inequality (1) of the main text. The total death rates of cells are as follows:

- for a one-drug treatment,

$$\begin{matrix} D_{0}=D_{1}=d, \\ D_{0*}=d+f_{1}(h_{1}), \end{matrix}$$

- for a two-drug treatment,

$$\begin{matrix} D_{00}=D_{01}=d+f_{2}(h_{1}), \\ D_{00*}=d+f_{2}(h_{1},h_{2}), \\ D_{11}=d, \end{matrix}$$

- for a the three-drug treatment,

$$\begin{matrix} D_{000}=D_{001}=d+f_{3}(h_{1},h_{2}), \\ D_{100}=d+f_{3}(h_{2},h_{3}), \\ D_{010}=d+f_{3}(h_{1},h_{3}), \\ D_{110}=d+f_{3}(h_{3}), \\ D_{101}=d+f_{3}(h_{2}), \\ D_{011}=d+f_{3}(h_{1}), \\ D_{000*}=D+f_{3}(h_{1},h_{2},h_{3}), \\ D_{111}=d. \end{matrix}$$

We note here that including the fully-susceptible type in the special model is equivalent to considering back-mutations. In principle, back-mutations (that is, mutations which return sensitivity to a given drug) can be formally included in all the models by drawing the double arrows between types, and writing down all the equations in the usual manner. However, it can be shown that under the assumption that the mutation rate (the probability to mutate per gene per cell division) is much smaller than the unity, back-mutations only introduce a small correction. Suppose that we have a diagram$x\to y\to z$, and start with *x* being of the order *1* (all cells susceptible). Then it is easy to see that *y* is of the order *u* and *z* is of the order$u^{2}$. Adding back-mutations (e.g. from *y* back to *x*) will introduce a correction of order *u* to *x*, and it will change *y* by a quantity of the order$u^{2}$. Both are negligible compared to the values in the model without back-mutations.

In the special model, we include back-mutations simply because they appear in the data (the number of mutants which confirm sensitivity to SGX70393). However, these mutations do not make a significant difference. With or without back-mutations, the model in the case of SGX70393 at small or medium concentration differs from the original model, because the wild type happens to be resistant, and we have to include a “special model” to account for that.

**3. Classification of resistant mutations**

The values *i_s_*, *i_sk_* , and *i_skm_* are calculated from the experimental data described and presented in Tables 1, 2.1-2.4 and 3.1-3.8.

In particular, the numbers of mutants resistant to single drugs, at different concentrations, are presented in Table 1. There, for each drug, we counted the number of mutations that may arise in a one-drug treatment at the corresponding concentration level of that drug. For example, to see how many mutants may arise in a treatment with imatinib at a low dose, we refer to the second column of table 2 of the main text (I/L) and count the number of rows marked “+”; we count 28 mutations and thus $i_{1}=28.$ For a treatment with SGX70393 at a low or medium dose, we refer to the second to last column of table 2 of the main text and we count all rows marked “+”; we count 29 mutations and write $i_{1}=29.$ The symbol “*” indicates that this drug cannot kill native (wild-type) cells; we need to account for the number of mutated cells that are susceptible to this drug (rows with no markings); we find 14 such mutations and write $i_{0}=14.$ The fact that the inhibitor SGX70393 at low and medium concentrations cannot kill wild-type cells presents a certain difficulty for the basic mathematical model described in Section 1. This difficulty is resolved in Section 2 where we create a special model to account for this case. The quantity $i_{0}$ is necessary for all calculations involving the special model.

Tables 2.1-2.4 identify the number of mutations that may arise in a two-drug combination treatment according to their class of concentration. There are three types of mutants in a two-drug treatment: ones that only confer resistance to the first drug, ones that only confer resistance to the second drug, and ones that confer cross-resistance to both drugs; the number of such mutants is $i_{1},$ $i_{2},$ and $i_{12},$ respectively. To identify these numbers, we first examine the columns of table 2 of the main text, pertaining to the drugs and their concentrations that are being considered. To find $i_{1},$ we count all rows that are marked “+” for the 1^st^ drug and unmarked for the 2^nd^ drug; to find $i_{2},$ we count all rows that are unmarked for the 1^st^ drug and marked “+” for the 2^nd^ drug; to find $i_{12},$ we count all rows that are marked “+” for both drugs. For example, consider a combination treatment with imatinib at low concentration as the 1^st^ drug and nilotinib at high concentration as the 2^nd^ drug. Then we must refer to the columns of table 2 of the main text marked I/L and N/H. We count the rows that are marked “+” for I/L and unmarked for N/H; we count 27 such rows and write $i_{1}=27.$ Next, we count the rows that are unmarked for I/L and marked “+” for N/H; we count 3 such rows and write $i_{2}=3.$ Finally, we count the rows that are marked “+” for both I/L and N/H; we count 1 row and write $i_{12}=1.$ Note that the number of mutations that confer resistance to drug 1 at its specific concentration will equal $i_{1}+i_{12};$ 28 mutants confer resistance to imatinib at low concentration (table 1 of the main text, row 2, column 2); in our example $i_{1}+i_{12}=27+1=28.$

The same method is used in the construction of tables 3.1-3.8 for three drug combination treatments. Since there are four inhibitors available at three different concentrations, we have 108 different possible treatments (4 different three-drug combinations, each drug with three different possible concentrations). Note that there are 7 different types of mutants, the counts of which are $i_{1},$ $i_{2},$ $i_{3},$ $i_{12},$ $i_{13},$ $i_{23},$ and $i_{123}.$ For example, consider a treatment with imatinib as the 1^st^ drug, nilotinib as the 2^nd^ drug, and SGX70393 as the 3^rd^ drug, all at high concentrations. Then we must consider columns I/H, N/H, and S/H in table 2 of the main text. Since there are no rows such that all three columns are marked “+”, we have $i_{123}=0.$ Similarly, there are no rows for which columns I/H and S/H, or N/H and S/H are marked “+”, so that $i_{13}=0,$ and $i_{23}=0,$ respectively. There is only one row where the columns I/H and N/H are both marked “+”, so that $i_{12}=1.$ The rest of the tables are constructed in a similar fashion.

| Category | **Imatinib**  Mutations | **Nilotinib**  Mutations | **Dasatinib**  Mutations | **SGX393**  Mutations |
| --- | --- | --- | --- | --- |
| Low | $i_{1}=$28 | $i_{1}=$11 | $i_{1}=$12 | $i_{0}=$14*, $i_{1}=$29 |
| Medium | $i_{1}=$13 | $i_{1}=$4 | $i_{1}=$7 | $i_{0}=$23*, $i_{1}=$20 |
| High | $i_{1}=$4 | $i_{1}=$1 | $i_{1}=$1 | $i_{1}=$6 |

**Table 1.** *Classification of the number of point mutations for each drug according to their category of concentration for a one drug treatment.* *This number is for the use in the special model and corresponds to the number of mutants susceptible to SGX393.

| **Category** | **Ima, Nilo**  Mutations | **Ima, Dasa**  Mutations | **Nilo, Dasa**  Mutations |
| --- | --- | --- | --- |
| L, L | | $i_{1}=$19 | | --- | | $i_{2}=$2 | | $i_{12}=$9 | | | $i_{1}=$19 | | --- | | $i_{2}=$3 | | $i_{12}=$9 | | | $i_{1}=$5 | | --- | | $i_{2}=$6 | | $i_{12}=$6 | |
| L, M | | $i_{1}=$24 | | --- | | $i_{2}=$0 | | $i_{12}=$4 | | | $i_{1}=$24 | | --- | | $i_{2}=$3 | | $i_{12}=$4 | | | $i_{1}=$10 | | --- | | $i_{2}=$6 | | $i_{12}=$1 | |
| M, L | | $i_{1}=$7 | | --- | | $i_{2}=$5 | | $i_{12}=$6 | | | $i_{1}=$6 | | --- | | $i_{2}=$5 | | $i_{12}=$7 | | | $i_{1}=$1 | | --- | | $i_{2}=$9 | | $i_{12}=$3 | |
| M, M | | $i_{1}=$9 | | --- | | $i_{2}=$0 | | $i_{12}=$4 | | | $i_{1}=$10 | | --- | | $i_{2}=$4 | | $i_{12}=$3 | | | $i_{1}=$3 | | --- | | $i_{2}=$6 | | $i_{12}=$1 | |

**Table 2.1.** *1 of 4* *classifications of the number of point mutations for each drug according to their category of concentration for a two drug combination treatment.*

| **Category** | **Ima, Nilo**  Mutations | **Ima, Dasa**  Mutations | **Nilo, Dasa**  Mutations |
| --- | --- | --- | --- |
| L, H | | $i_{1}=$27 | | --- | | $i_{2}=$3 | | $i_{12}=$1 | | | $i_{1}=$27 | | --- | | $i_{2}=$0 | | $i_{12}=$1 | | | $i_{1}=$10 | | --- | | $i_{2}=$0 | | $i_{12}=$1 | |
| H, L | | $i_{1}=$1 | | --- | | $i_{2}=$8 | | $i_{12}=$3 | | | $i_{1}=$1 | | --- | | $i_{2}=$9 | | $i_{12}=$3 | | | $i_{1}=$0 | | --- | | $i_{2}=$11 | | $i_{12}=$1 | |
| M, H | | $i_{1}=$12 | | --- | | $i_{2}=$0 | | $i_{12}=$1 | | | $i_{1}=$12 | | --- | | $i_{2}=$0 | | $i_{12}=$1 | | | $i_{1}=$3 | | --- | | $i_{2}=$0 | | $i_{12}=$1 | |
| H, M | | $i_{1}=$2 | | --- | | $i_{2}=$2 | | $i_{12}=$2 | | | $i_{1}=$2 | | --- | | $i_{2}=$5 | | $i_{12}=$2 | | | $i_{1}=$0 | | --- | | $i_{2}=$6 | | $i_{12}=$1 | |
| H, H | | $i_{1}=$3 | | --- | | $i_{2}=$0 | | $i_{12}=$1 | | | $i_{1}=$3 | | --- | | $i_{2}=$0 | | $i_{12}=$1 | | | $i_{1}=$0 | | --- | | $i_{2}=$0 | | $i_{12}=$1 | |

**Table 2.2.** *2 of 4* *classifications of the number of point mutations for each drug according to their category of concentration for a two drug combination treatment.*

| **Category** | **Ima, SGX**  Mutations | **Nilo, SGX**  Mutations | **Dasa, SGX**  Mutations |
| --- | --- | --- | --- |
| L, L | | $i_{0}=$3* | | --- | | $i_{1}=$11 | | $i_{2}=$12 | | $i_{12}=$17 | | | $i_{0}=$9* | | --- | | $i_{1}=$5 | | $i_{2}=$23 | | $i_{12}=$6 | | | $i_{0}=$12* | | --- | | $i_{1}=$2 | | $i_{2}=$19 | | $i_{12}=$10 | |
| L, M | | $i_{0}=$9* | | --- | | $i_{1}=$14 | | $i_{2}=$6 | | $i_{12}=$14 | | | $i_{0}=$17* | | --- | | $i_{1}=$6 | | $i_{2}=$15 | | $i_{12}=$5 | | | $i_{0}=$20* | | --- | | $i_{1}=$3 | | $i_{2}=$11 | | $i_{12}=$9 | |
| M, L | | $i_{0}=$12* | | --- | | $i_{1}=$2 | | $i_{2}=$18 | | $i_{12}=$11 | | | $i_{0}=$13* | | --- | | $i_{1}=$1 | | $i_{2}=$26 | | $i_{12}=$3 | | | $i_{0}=$12* | | --- | | $i_{1}=$2 | | $i_{2}=$24 | | $i_{12}=$5 | |
| M, M | | $i_{0}=$21* | | --- | | $i_{1}=$2 | | $i_{2}=$9 | | $i_{12}=$11 | | | $i_{0}=$22* | | --- | | $i_{1}=$1 | | $i_{2}=$17 | | $i_{12}=$3 | | | $i_{0}=$21* | | --- | | $i_{1}=$2 | | $i_{2}=$15 | | $i_{12}=$5 | |

**Table 2.3.** *3 of 4* *classifications of the number of point mutations for each drug according to their category of concentration for a two drug combination treatment.* *This number is for the use in the special model and indicates the number of mutants susceptible to the given drug combination.

| **Category** | **Ima, SGX**  Mutations | **Nilo, SGX**  Mutations | **Dasa, SGX**  Mutations |
| --- | --- | --- | --- |
| L, H | | $i_{1}=$23 | | --- | | $i_{2}=$1 | | $i_{12}=$5 | | | $i_{1}=$8 | | --- | | $i_{2}=$3 | | $i_{12}=$3 | | | $i_{1}=$8 | | --- | | $i_{2}=$2 | | $i_{12}=$4 | |
| H, L | | $i_{0}=$13* | | --- | | $i_{1}=$1 | | $i_{2}=$26 | | $i_{12}=$3 | | | $i_{0}=$13* | | --- | | $i_{1}=$1 | | $i_{2}=$29 | | $i_{12}=$0 | | | $i_{0}=$13* | | --- | | $i_{1}=$1 | | $i_{2}=$29 | | $i_{12}=$0 | |
| M, H | | $i_{1}=$8 | | --- | | $i_{2}=$1 | | $i_{12}=$5 | | | $i_{1}=$2 | | --- | | $i_{2}=$4 | | $i_{12}=$2 | | | $i_{1}=$6 | | --- | | $i_{2}=$5 | | $i_{12}=$1 | |
| H, M | | $i_{0}=$22* | | --- | | $i_{1}=$1 | | $i_{2}=$17 | | $i_{12}=$3 | | | $i_{0}=$22* | | --- | | $i_{1}=$1 | | $i_{2}=$20 | | $i_{12}=$0 | | | $i_{0}=$22* | | --- | | $i_{1}=$1 | | $i_{2}=$20 | | $i_{12}=$0 | |
| H, H | | $i_{1}=$4 | | --- | | $i_{2}=$6 | | $i_{12}=$0 | | | $i_{1}=$1 | | --- | | $i_{2}=$6 | | $i_{12}=$0 | | | $i_{1}=$1 | | --- | | $i_{2}=$6 | | $i_{12}=$0 | |

**Table 2.4.** *4 of 4* *classifications of the number of point mutations for each drug according to their category of concentration for a two drug combination treatment.* *This number is for the use in the special model and indicates the number of mutants susceptible to the given drug combination.

| **Category** | **Ima, Nilo, Dasa**  Mutations | **Ima, Nilo, SGX**  Mutations |
| --- | --- | --- |
| L, L, L | | $i_{1}=$16 | $i_{2}=$2 | | --- | --- | | $i_{3}=$3 | $i_{12}=$3 | | $i_{13}=$3 | $i_{23}=$0 | | $i_{123}=$6 |  | | | $i_{1}=$8 | $i_{2}=$2 | | --- | --- | | $i_{3}=$12 | $i_{12}=$3 | | $i_{13}=$11 | $i_{23}=$0 | | $i_{123}=$6 | $i_{0}=$1* | |
| M, L, L | | $i_{1}=$5 | $i_{2}=$4 | | --- | --- | | $i_{3}=$4 | $i_{12}=$1 | | $i_{13}=$2 | $i_{23}=$1 | | $i_{123}=$5 |  | | | $i_{1}=$1 | $i_{2}=$4 | | --- | --- | | $i_{3}=$17 | $i_{12}=$1 | | $i_{13}=$6 | $i_{23}=$1 | | $i_{123}=$5 | $i_{0}=$8* | |
| L, M, L | | $i_{1}=$18 | $i_{2}=$0 | | --- | --- | | $i_{3}=$3 | $i_{12}=$1 | | $i_{13}=$6 | $i_{23}=$0 | | $i_{123}=$3 |  | | | $i_{1}=$10 | $i_{2}=$0 | | --- | --- | | $i_{3}=$12 | $i_{12}=$1 | | $i_{13}=$14 | $i_{23}=$0 | | $i_{123}=$3 | $i_{0}=$3* | |
| L, L, M | | $i_{1}=$16 | $i_{2}=$2 | | --- | --- | | $i_{3}=$3 | $i_{12}=$8 | | $i_{13}=$3 | $i_{23}=$0 | | $i_{123}=$1 |  | | | $i_{1}=$10 | $i_{2}=$2 | | --- | --- | | $i_{3}=$6 | $i_{12}=$4 | | $i_{13}=$9 | $i_{23}=$0 | | $i_{123}=$5 | $i_{0}=$7* | |
| H, L, L | | $i_{1}=$0 | $i_{2}=$4 | | --- | --- | | $i_{3}=$5 | $i_{12}=$1 | | $i_{13}=$1 | $i_{23}=$4 | | $i_{123}=$2 |  | | | $i_{1}=$0 | $i_{2}=$4 | | --- | --- | | $i_{3}=$22 | $i_{12}=$1 | | $i_{13}=$1 | $i_{23}=$4 | | $i_{123}=$2 | $i_{0}=$9* | |
| L, H, L | | $i_{1}=$19 | $i_{2}=$0 | | --- | --- | | $i_{3}=$3 | $i_{12}=$0 | | $i_{13}=$8 | $i_{23}=$0 | | $i_{123}=$1 |  | | | $i_{1}=$10 | $i_{2}=$0 | | --- | --- | | $i_{3}=$12 | $i_{12}=$1 | | $i_{13}=$17 | $i_{23}=$0 | | $i_{123}=$0 | $i_{0}=$3* | |
| L, L, H | | $i_{1}=$19 | $i_{2}=$2 | | --- | --- | | $i_{3}=$0 | $i_{12}=$8 | | $i_{13}=$0 | $i_{23}=$0 | | $i_{123}=$1 |  | | | $i_{1}=$17 | $i_{2}=$2 | | --- | --- | | $i_{3}=$1 | $i_{12}=$6 | | $i_{13}=$2 | $i_{23}=$0 | | $i_{123}=$3 |  | |

**Table 3.1.** *1 of 8 classifications of the number of point mutations for each drug according to their category of concentration for a three drug combination treatment.*  *This number is for the use in the special model and indicates the number of mutants susceptible to the given drug combination.

| **Category** | **Ima, Nilo, Dasa**  Mutations | **Ima, Nilo, SGX**  Mutations |
| --- | --- | --- |
| M, M, M | | $i_{1}=$7 | $i_{2}=$0 | | --- | --- | | $i_{3}=$4 | $i_{12}=$3 | | $i_{13}=$2 | $i_{23}=$0 | | $i_{123}=$1 |  | | | $i_{1}=$1 | $i_{2}=$0 | | --- | --- | | $i_{3}=$9 | $i_{12}=$1 | | $i_{13}=$8 | $i_{23}=$0 | | $i_{123}=$3 | $i_{0}=$21* | |
| H, L, M | | $i_{1}=$0 | $i_{2}=$8 | | --- | --- | | $i_{3}=$5 | $i_{12}=$2 | | $i_{13}=$1 | $i_{23}=$0 | | $i_{123}=$1 |  | | | $i_{1}=$0 | $i_{2}=$5 | | --- | --- | | $i_{3}=$14 | $i_{12}=$1 | | $i_{13}=$1 | $i_{23}=$3 | | $i_{123}=$2 | $i_{0}=$17* | |
| H, M, L | | $i_{1}=$0 | $i_{2}=$0 | | --- | --- | | $i_{3}=$7 | $i_{12}=$1 | | $i_{13}=$2 | $i_{23}=$2 | | $i_{123}=$1 |  | | | $i_{1}=$0 | $i_{2}=$0 | | --- | --- | | $i_{3}=$24 | $i_{12}=$1 | | $i_{13}=$2 | $i_{23}=$2 | | $i_{123}=$1 | $i_{0}=$13* | |
| M, H, L | | $i_{1}=$6 | $i_{2}=$0 | | --- | --- | | $i_{3}=$5 | $i_{12}=$0 | | $i_{13}=$6 | $i_{23}=$0 | | $i_{123}=$1 |  | | | $i_{1}=$1 | $i_{2}=$0 | | --- | --- | | $i_{3}=$18 | $i_{12}=$1 | | $i_{13}=$11 | $i_{23}=$0 | | $i_{123}=$0 | $i_{0}=$12* | |
| M, L, H | | $i_{1}=$7 | $i_{2}=$5 | | --- | --- | | $i_{3}=$0 | $i_{12}=$5 | | $i_{13}=$0 | $i_{23}=$0 | | $i_{123}=$1 |  | | | $i_{1}=$5 | $i_{2}=$5 | | --- | --- | | $i_{3}=$1 | $i_{12}=$3 | | $i_{13}=$2 | $i_{23}=$0 | | $i_{123}=$3 |  | |
| L, H, M | | $i_{1}=$24 | $i_{2}=$0 | | --- | --- | | $i_{3}=$3 | $i_{12}=$0 | | $i_{13}=$3 | $i_{23}=$0 | | $i_{123}=$1 |  | | | $i_{1}=$13 | $i_{2}=$0 | | --- | --- | | $i_{3}=$6 | $i_{12}=$1 | | $i_{13}=$14 | $i_{23}=$0 | | $i_{123}=$0 | $i_{0}=$9* | |
| L, M, H | | $i_{1}=$24 | $i_{2}=$0 | | --- | --- | | $i_{3}=$0 | $i_{12}=$3 | | $i_{13}=$0 | $i_{23}=$0 | | $i_{123}=$1 |  | | | $i_{1}=$21 | $i_{2}=$0 | | --- | --- | | $i_{3}=$1 | $i_{12}=$2 | | $i_{13}=$3 | $i_{23}=$0 | | $i_{123}=$2 |  | |

**Table 3.2.** *2 of 8 classifications of the number of point mutations for each drug according to their category of concentration for a three drug combination treatment.*  *This number is for the use in the special model and indicates the number of mutants susceptible to the given drug combination.

| **Category** | **Ima, Nilo, Dasa**  Mutations | **Ima, Nilo, SGX**  Mutations |
| --- | --- | --- |
| L, M, M | | $i_{1}=$21 | $i_{2}=$0 | | --- | --- | | $i_{3}=$3 | $i_{12}=$3 | | $i_{13}=$3 | $i_{23}=$0 | | $i_{123}=$1 |  | | | $i_{1}=$13 | $i_{2}=$0 | | --- | --- | | $i_{3}=$6 | $i_{12}=$1 | | $i_{13}=$11 | $i_{23}=$0 | | $i_{123}=$3 | $i_{0}=$9* | |
| M, L, M | | $i_{1}=$5 | $i_{2}=$5 | | --- | --- | | $i_{3}=$4 | $i_{12}=$5 | | $i_{13}=$2 | $i_{23}=$0 | | $i_{123}=$1 |  | | | $i_{1}=$1 | $i_{2}=$5 | | --- | --- | | $i_{3}=$9 | $i_{12}=$1 | | $i_{13}=$6 | $i_{23}=$0 | | $i_{123}=$5 | $i_{0}=$16* | |
| M, M, L | | $i_{1}=$5 | $i_{2}=$0 | | --- | --- | | $i_{3}=$5 | $i_{12}=$1 | | $i_{13}=$4 | $i_{23}=$0 | | $i_{123}=$3 |  | | | $i_{1}=$1 | $i_{2}=$0 | | --- | --- | | $i_{3}=$18 | $i_{12}=$1 | | $i_{13}=$8 | $i_{23}=$0 | | $i_{123}=$3 | $i_{0}=$12* | |
| H, M, M | | $i_{1}=$1 | $i_{2}=$2 | | --- | --- | | $i_{3}=$5 | $i_{12}=$1 | | $i_{13}=$1 | $i_{23}=$0 | | $i_{123}=$1 |  | | | $i_{1}=$0 | $i_{2}=$0 | | --- | --- | | $i_{3}=$15 | $i_{12}=$1 | | $i_{13}=$2 | $i_{23}=$2 | | $i_{123}=$1 | $i_{0}=$22* | |
| M, H, M | | $i_{1}=$10 | $i_{2}=$0 | | --- | --- | | $i_{3}=$4 | $i_{12}=$0 | | $i_{13}=$2 | $i_{23}=$0 | | $i_{123}=$1 |  | | | $i_{1}=$1 | $i_{2}=$0 | | --- | --- | | $i_{3}=$9 | $i_{12}=$1 | | $i_{13}=$11 | $i_{23}=$0 | | $i_{123}=$0 | $i_{0}=$21* | |
| M, M, H | | $i_{1}=$9 | $i_{2}=$0 | | --- | --- | | $i_{3}=$0 | $i_{12}=$3 | | $i_{13}=$0 | $i_{23}=$0 | | $i_{123}=$1 |  | | | $i_{1}=$6 | $i_{2}=$0 | | --- | --- | | $i_{3}=$1 | $i_{12}=$2 | | $i_{13}=$3 | $i_{23}=$0 | | $i_{123}=$2 |  | |

**Table 3.3.** *3 of 8 classifications of the number of point mutations for each drug according to their category of concentration for a three drug combination treatment.*  *This number is for the use in the special model and indicates the number of mutants susceptible to the given drug combination.

| **Category** | **Ima, Nilo, Dasa**  Mutations | **Ima, Nilo, SGX**  Mutations |
| --- | --- | --- |
| L, H, H | | $i_{1}=$27 | $i_{2}=$0 | | --- | --- | | $i_{3}=$0 | $i_{12}=$0 | | $i_{13}=$0 | $i_{23}=$0 | | $i_{123}=$1 |  | | | $i_{1}=$22 | $i_{2}=$0 | | --- | --- | | $i_{3}=$1 | $i_{12}=$1 | | $i_{13}=$5 | $i_{23}=$0 | | $i_{123}=$0 |  | |
| H, L, H | | $i_{1}=$1 | $i_{2}=$8 | | --- | --- | | $i_{3}=$0 | $i_{12}=$2 | | $i_{13}=$0 | $i_{23}=$0 | | $i_{123}=$1 |  | | | $i_{1}=$1 | $i_{2}=$5 | | --- | --- | | $i_{3}=$3 | $i_{12}=$3 | | $i_{13}=$0 | $i_{23}=$3 | | $i_{123}=$0 |  | |
| H, H, L | | $i_{1}=$1 | $i_{2}=$0 | | --- | --- | | $i_{3}=$9 | $i_{12}=$0 | | $i_{13}=$2 | $i_{23}=$0 | | $i_{123}=$1 |  | | | $i_{1}=$0 | $i_{2}=$0 | | --- | --- | | $i_{3}=$26 | $i_{12}=$1 | | $i_{13}=$3 | $i_{23}=$0 | | $i_{123}=$0 | $i_{0}=$13* | |
| M, H, H | | $i_{1}=$12 | $i_{2}=$0 | | --- | --- | | $i_{3}=$0 | $i_{12}=$0 | | $i_{13}=$0 | $i_{23}=$0 | | $i_{123}=$1 |  | | | $i_{1}=$7 | $i_{2}=$0 | | --- | --- | | $i_{3}=$1 | $i_{12}=$1 | | $i_{13}=$5 | $i_{23}=$0 | | $i_{123}=$0 |  | |
| H, M, H | | $i_{1}=$2 | $i_{2}=$2 | | --- | --- | | $i_{3}=$0 | $i_{12}=$1 | | $i_{13}=$0 | $i_{23}=$0 | | $i_{123}=$1 |  | | | $i_{1}=$2 | $i_{2}=$0 | | --- | --- | | $i_{3}=$4 | $i_{12}=$2 | | $i_{13}=$0 | $i_{23}=$2 | | $i_{123}=$0 |  | |
| H, H, M | | $i_{1}=$2 | $i_{2}=$0 | | --- | --- | | $i_{3}=$5 | $i_{12}=$0 | | $i_{13}=$1 | $i_{23}=$0 | | $i_{123}=$1 |  | | | $i_{1}=$0 | $i_{2}=$0 | | --- | --- | | $i_{3}=$17 | $i_{12}=$1 | | $i_{13}=$3 | $i_{23}=$0 | | $i_{123}=$0 | $i_{0}=$22* | |
| H, H, H | | $i_{1}=$3 | $i_{2}=$0 | | --- | --- | | $i_{3}=$0 | $i_{12}=$0 | | $i_{13}=$0 | $i_{23}=$0 | | $i_{123}=$1 |  | | | $i_{1}=$3 | $i_{2}=$0 | | --- | --- | | $i_{3}=$6 | $i_{12}=$1 | | $i_{13}=$0 | $i_{23}=$0 | | $i_{123}=$0 |  | |

**Table 3.4.** *4 of 8 classifications of the number of point mutations for each drug according to their category of concentration for a three drug combination treatment.*  *This number is for the use in the special model and indicates the number of mutants susceptible to the given drug combination.

| **Category** | **Ima, Dasa, SGX**  Mutations | **Nilo, Dasa, SGX**  Mutations |
| --- | --- | --- |
| L, L, L | | $i_{1}=$10 | $i_{2}=$1 | | --- | --- | | $i_{3}=$10 | $i_{12}=$1 | | $i_{13}=$9 | $i_{23}=$2 | | $i_{123}=$8 | $i_{0}=$2* | | | $i_{1}=$4 | $i_{2}=$1 | | --- | --- | | $i_{3}=$18 | $i_{12}=$1 | | $i_{13}=$1 | $i_{23}=$5 | | $i_{123}=$5 | $i_{0}=$8* | |
| M, L, L | | $i_{1}=$1 | $i_{2}=$1 | | --- | --- | | $i_{3}=$14 | $i_{12}=$1 | | $i_{13}=$5 | $i_{23}=$4 | | $i_{123}=$6 | $i_{0}=$11* | | | $i_{1}=$0 | $i_{2}=$1 | | --- | --- | | $i_{3}=$18 | $i_{12}=$1 | | $i_{13}=$1 | $i_{23}=$8 | | $i_{123}=$2 | $i_{0}=$12* | |
| L, M, L | | $i_{1}=$10 | $i_{2}=$1 | | --- | --- | | $i_{3}=$10 | $i_{12}=$1 | | $i_{13}=$14 | $i_{23}=$2 | | $i_{123}=$3 | $i_{0}=$2* | | | $i_{1}=$4 | $i_{2}=$1 | | --- | --- | | $i_{3}=$18 | $i_{12}=$1 | | $i_{13}=$6 | $i_{23}=$5 | | $i_{123}=$0 | $i_{0}=$8* | |
| L, L, M | | $i_{1}=$12 | $i_{2}=$1 | | --- | --- | | $i_{3}=$4 | $i_{12}=$2 | | $i_{13}=$7 | $i_{23}=$2 | | $i_{123}=$7 | $i_{0}=$8* | | | $i_{1}=$4 | $i_{2}=$1 | | --- | --- | | $i_{3}=$10 | $i_{12}=$2 | | $i_{13}=$1 | $i_{23}=$5 | | $i_{123}=$4 | $i_{0}=$16* | |
| H, L, L | | $i_{1}=$0 | $i_{2}=$1 | | --- | --- | | $i_{3}=$18 | $i_{12}=$1 | | $i_{13}=$1 | $i_{23}=$8 | | $i_{123}=$2 | $i_{0}=$12* | | | $i_{1}=$0 | $i_{2}=$1 | | --- | --- | | $i_{3}=$19 | $i_{12}=$1 | | $i_{13}=$0 | $i_{23}=$10 | | $i_{123}=$0 | $i_{0}=$12* | |
| L, H, L | | $i_{1}=$10 | $i_{2}=$0 | | --- | --- | | $i_{3}=$12 | $i_{12}=$1 | | $i_{13}=$17 | $i_{23}=$0 | | $i_{123}=$0 | $i_{0}=$3* | | | $i_{1}=$4 | $i_{2}=$0 | | --- | --- | | $i_{3}=$23 | $i_{12}=$1 | | $i_{13}=$6 | $i_{23}=$0 | | $i_{123}=$0 | $i_{0}=$9* | |
| L, L, H | | $i_{1}=$17 | $i_{2}=$2 | | --- | --- | | $i_{3}=$0 | $i_{12}=$6 | | $i_{13}=$2 | $i_{23}=$1 | | $i_{123}=$3 |  | | | $i_{1}=$5 | $i_{2}=$5 | | --- | --- | | $i_{3}=$2 | $i_{12}=$3 | | $i_{13}=$0 | $i_{23}=$1 | | $i_{123}=$3 |  | |

**Table 3.5.** *5 of 8 classifications of the number of point mutations for each drug according to their category of concentration for a three drug combination treatment.*  *This number is for the use in the special model and indicates the number of mutants susceptible to the given drug combination.

| **Category** | **Ima, Dasa, SGX**  Mutations | **Nilo, Dasa, SGX**  Mutations |
| --- | --- | --- |
| M, M, M | | $i_{1}=$1 | $i_{2}=$1 | | --- | --- | | $i_{3}=$6 | $i_{12}=$1 | | $i_{13}=$9 | $i_{23}=$3 | | $i_{123}=$2 | $i_{0}=$20* | | | $i_{1}=$0 | $i_{2}=$1 | | --- | --- | | $i_{3}=$12 | $i_{12}=$1 | | $i_{13}=$3 | $i_{23}=$5 | | $i_{123}=$0 | $i_{0}=$21* | |
| H, L, M | | $i_{1}=$0 | $i_{2}=$2 | | --- | --- | | $i_{3}=$10 | $i_{12}=$1 | | $i_{13}=$1 | $i_{23}=$7 | | $i_{123}=$2 | $i_{0}=$20* | | | $i_{1}=$0 | $i_{2}=$2 | | --- | --- | | $i_{3}=$11 | $i_{12}=$1 | | $i_{13}=$0 | $i_{23}=$9 | | $i_{123}=$0 | $i_{0}=$20* | |
| H, M, L | | $i_{1}=$0 | $i_{2}=$1 | | --- | --- | | $i_{3}=$22 | $i_{12}=$1 | | $i_{13}=$2 | $i_{23}=$4 | | $i_{123}=$1 | $i_{0}=$12* | | | $i_{1}=$0 | $i_{2}=$1 | | --- | --- | | $i_{3}=$24 | $i_{12}=$1 | | $i_{13}=$0 | $i_{23}=$5 | | $i_{123}=$0 | $i_{0}=$12* | |
| M, H, L | | $i_{1}=$1 | $i_{2}=$0 | | --- | --- | | $i_{3}=$18 | $i_{12}=$1 | | $i_{13}=$11 | $i_{23}=$0 | | $i_{123}=$0 | $i_{0}=$12* | | | $i_{1}=$0 | $i_{2}=$0 | | --- | --- | | $i_{3}=$26 | $i_{12}=$1 | | $i_{13}=$3 | $i_{23}=$0 | | $i_{123}=$0 | $i_{0}=$13* | |
| M, L, H | | $i_{1}=$4 | $i_{2}=$4 | | --- | --- | | $i_{3}=$0 | $i_{12}=$4 | | $i_{13}=$2 | $i_{23}=$1 | | $i_{123}=$3 |  | | | $i_{1}=$1 | $i_{2}=$7 | | --- | --- | | $i_{3}=$2 | $i_{12}=$1 | | $i_{13}=$0 | $i_{23}=$2 | | $i_{123}=$2 |  | |
| L, H, M | | $i_{1}=$13 | $i_{2}=$0 | | --- | --- | | $i_{3}=$6 | $i_{12}=$1 | | $i_{13}=$14 | $i_{23}=$0 | | $i_{123}=$0 | $i_{0}=$9* | | | $i_{1}=$5 | $i_{2}=$0 | | --- | --- | | $i_{3}=$15 | $i_{12}=$1 | | $i_{13}=$5 | $i_{23}=$0 | | $i_{123}=$0 | $i_{0}=$17* | |
| L, M, H | | $i_{1}=$19 | $i_{2}=$2 | | --- | --- | | $i_{3}=$0 | $i_{12}=$4 | | $i_{13}=$5 | $i_{23}=$1 | | $i_{123}=$0 |  | | | $i_{1}=$7 | $i_{2}=$5 | | --- | --- | | $i_{3}=$2 | $i_{12}=$1 | | $i_{13}=$3 | $i_{23}=$1 | | $i_{123}=$0 |  | |

**Table 3.6.** *6 of 8 classifications of the number of point mutations for each drug according to their category of concentration for a three drug combination treatment.*  *This number is for the use in the special model and indicates the number of mutants susceptible to the given drug combination.

| **Category** | **Ima, Dasa, SGX**  Mutations | **Nilo, Dasa, SGX**  Mutations |
| --- | --- | --- |
| L, M, M | | $i_{1}=$13 | $i_{2}=$1 | | --- | --- | | $i_{3}=$4 | $i_{12}=$1 | | $i_{13}=$11 | $i_{23}=$2 | | $i_{123}=$3 | $i_{0}=$8* | | | $i_{1}=$5 | $i_{2}=$1 | | --- | --- | | $i_{3}=$10 | $i_{12}=$1 | | $i_{13}=$5 | $i_{23}=$5 | | $i_{123}=$0 | $i_{0}=$16* | |
| M, L, M | | $i_{1}=$1 | $i_{2}=$2 | | --- | --- | | $i_{3}=$6 | $i_{12}=$1 | | $i_{13}=$5 | $i_{23}=$3 | | $i_{123}=$6 | $i_{0}=$19* | | | $i_{1}=$0 | $i_{2}=$2 | | --- | --- | | $i_{3}=$10 | $i_{12}=$1 | | $i_{13}=$1 | $i_{23}=$7 | | $i_{123}=$2 | $i_{0}=$20* | |
| M, M, L | | $i_{1}=$1 | $i_{2}=$1 | | --- | --- | | $i_{3}=$15 | $i_{12}=$1 | | $i_{13}=$9 | $i_{23}=$3 | | $i_{123}=$2 | $i_{0}=$11* | | | $i_{1}=$0 | $i_{2}=$1 | | --- | --- | | $i_{3}=$21 | $i_{12}=$1 | | $i_{13}=$3 | $i_{23}=$5 | | $i_{123}=$0 | $i_{0}=$12* | |
| H, M, M | | $i_{1}=$0 | $i_{2}=$1 | | --- | --- | | $i_{3}=$13 | $i_{12}=$1 | | $i_{13}=$2 | $i_{23}=$4 | | $i_{123}=$1 | $i_{0}=$21* | | | $i_{1}=$0 | $i_{2}=$1 | | --- | --- | | $i_{3}=$15 | $i_{12}=$1 | | $i_{13}=$0 | $i_{23}=$5 | | $i_{123}=$0 | $i_{0}=$21* | |
| M, H, M | | $i_{1}=$1 | $i_{2}=$0 | | --- | --- | | $i_{3}=$9 | $i_{12}=$1 | | $i_{13}=$11 | $i_{23}=$0 | | $i_{123}=$0 | $i_{0}=$21* | | | $i_{1}=$0 | $i_{2}=$0 | | --- | --- | | $i_{3}=$17 | $i_{12}=$1 | | $i_{13}=$3 | $i_{23}=$0 | | $i_{123}=$0 | $i_{0}=$22* | |
| M, M, H | | $i_{1}=$5 | $i_{2}=$3 | | --- | --- | | $i_{3}=$0 | $i_{12}=$3 | | $i_{13}=$5 | $i_{23}=$1 | | $i_{123}=$0 |  | | | $i_{1}=$1 | $i_{2}=$5 | | --- | --- | | $i_{3}=$3 | $i_{12}=$1 | | $i_{13}=$2 | $i_{23}=$1 | | $i_{123}=$0 |  | |

**Table 3.7.** *7 of 8 classifications of the number of point mutations for each drug according to their category of concentration for a three drug combination treatment.*  *This number is for the use in the special model and indicates the number of mutants susceptible to the given drug combination.

| **Category** | **Ima, Dasa, SGX**  Mutations | **Nilo, Dasa, SGX**  Mutations |
| --- | --- | --- |
| L, H, H | | $i_{1}=$22 | $i_{2}=$0 | | --- | --- | | $i_{3}=$1 | $i_{12}=$1 | | $i_{13}=$5 | $i_{23}=$0 | | $i_{123}=$0 |  | | | $i_{1}=$7 | $i_{2}=$0 | | --- | --- | | $i_{3}=$3 | $i_{12}=$1 | | $i_{13}=$3 | $i_{23}=$0 | | $i_{123}=$0 |  | |
| H, L, H | | $i_{1}=$1 | $i_{2}=$5 | | --- | --- | | $i_{3}=$2 | $i_{12}=$3 | | $i_{13}=$0 | $i_{23}=$4 | | $i_{123}=$0 |  | | | $i_{1}=$0 | $i_{2}=$7 | | --- | --- | | $i_{3}=$2 | $i_{12}=$1 | | $i_{13}=$0 | $i_{23}=$4 | | $i_{123}=$0 |  | |
| H, H, L | | $i_{1}=$0 | $i_{2}=$0 | | --- | --- | | $i_{3}=$26 | $i_{12}=$1 | | $i_{13}=$3 | $i_{23}=$0 | | $i_{123}=$0 | $i_{0}=$13* | | | $i_{1}=$0 | $i_{2}=$0 | | --- | --- | | $i_{3}=$28 | $i_{12}=$1 | | $i_{13}=$0 | $i_{23}=$0 | | $i_{123}=$0 | $i_{0}=$14* | |
| M, H, H | | $i_{1}=$7 | $i_{2}=$0 | | --- | --- | | $i_{3}=$1 | $i_{12}=$1 | | $i_{13}=$5 | $i_{23}=$0 | | $i_{123}=$0 |  | | | $i_{1}=$1 | $i_{2}=$0 | | --- | --- | | $i_{3}=$4 | $i_{12}=$1 | | $i_{13}=$2 | $i_{23}=$0 | | $i_{123}=$0 |  | |
| H, M, H | | $i_{1}=$2 | $i_{2}=$4 | | --- | --- | | $i_{3}=$5 | $i_{12}=$2 | | $i_{13}=$0 | $i_{23}=$1 | | $i_{123}=$0 |  | | | $i_{1}=$0 | $i_{2}=$5 | | --- | --- | | $i_{3}=$5 | $i_{12}=$1 | | $i_{13}=$0 | $i_{23}=$1 | | $i_{123}=$0 |  | |
| H, H, M | | $i_{1}=$0 | $i_{2}=$0 | | --- | --- | | $i_{3}=$17 | $i_{12}=$1 | | $i_{13}=$3 | $i_{23}=$0 | | $i_{123}=$0 | $i_{0}=$22* | | | $i_{1}=$0 | $i_{2}=$0 | | --- | --- | | $i_{3}=$20 | $i_{12}=$1 | | $i_{13}=$0 | $i_{23}=$0 | | $i_{123}=$0 | $i_{0}=$22* | |
| H, H, H | | $i_{1}=$3 | $i_{2}=$0 | | --- | --- | | $i_{3}=$6 | $i_{12}=$1 | | $i_{13}=$0 | $i_{23}=$0 | | $i_{123}=$0 |  | | | $i_{1}=$0 | $i_{2}=$0 | | --- | --- | | $i_{3}=$6 | $i_{12}=$1 | | $i_{13}=$0 | $i_{23}=$0 | | $i_{123}=$0 |  | |

**Table 3.8.** *8 of 8 classifications of the number of point mutations for each drug according to their category of concentration for a three drug combination treatment.* *This number is for the use in the special model and indicates the number of mutants susceptible to the given drug combination.
